# The Predicted ABC Transporter AbcEDCBA Is Required for Type IV Secretion System Expression and Lysosomal Evasion by *Brucella ovis*

**DOI:** 10.1371/journal.pone.0114532

**Published:** 2014-12-04

**Authors:** Teane M. A. Silva, Juliana P. S. Mol, Maria G. Winter, Vidya Atluri, Mariana N. Xavier, Simone F. Pires, Tatiane A. Paixão, Hélida M. Andrade, Renato L. Santos, Renee M. Tsolis

**Affiliations:** 1 Departamento de Clínica e Cirurgia Veterinária, Escola de Veterinária da Universidade Federal de Minas Gerais, Belo Horizonte, Minas Gerais, Brazil; 2 Department of Medical Microbiology and Immunology, University of California Davis, Davis, California, United States of America; 3 Departamento de Patologia Geral, Instituto de Ciências Biológicas da Universidade Federal de Minas Gerais, Belo Horizonte, Minas Gerais, Brazil; 4 Departamento de Parasitologia, Instituto de Ciências Biológicas da Universidade Federal de Minas Gerais, Belo Horizonte, Minas Gerais, Brazil; University of Arkansas for Medical Sciences, United States of America

## Abstract

*Brucella ovis* is a major cause of reproductive failure in rams and it is one of the few well-described *Brucella* species that is not zoonotic. Previous work showed that a *B. ovis* mutant lacking a species-specific ABC transporter (Δ*abcBA*) was attenuated in mice and was unable to survive in macrophages. The aim of this study was to evaluate the role of this ABC transporter during intracellular survival of *B. ovis*. In HeLa cells, *B. ovis* WT was able to survive and replicate at later time point (48 hpi), whereas an Δ*abcBA* mutant was attenuated at 24 hpi. The reduced survival of the Δ*abcBA* mutant was associated with a decreased ability to exclude the lysosomal marker LAMP1 from its vacuolar membrane, suggesting a failure to establish a replicative niche. The Δ*abcBA* mutant showed a reduced abundance of the Type IV secretion system (T4SS) proteins VirB8 and VirB11 in both rich and acid media, when compared to WT *B. ovis*. However, mRNA levels of *virB1*, *virB8*, *hutC,* and *vjbR* were similar in both strains. These results support the notion that the ABC transporter encoded by *abcEDCBA* or its transported substrate acts at a post-transcriptional level to promote the optimal expression of the *B. ovis* T4SS within infected host cells.

## Introduction

*Brucella ovis* is one of the main causes of reproductive failure in sheep [1]. In sexually mature rams, the infection causes chronic epididymitis, orchitis, and infertility, whereas in ewes, it is characterized by uncommon abortion and stillbirth [2], [3]. *B. ovis* has a worldwide distribution in main sheep-raising areas, resulting in significant economic losses for the sheep industry [1], [4]. This organism is a stably rough Gram-negative coccobacillus that belongs to the alpha-2-Proteobacteria family [2], [5]. Unlike most of the well-described *Brucella* spp., *B. ovis* does not cause disease in humans [2].

Similar to other *Brucella* spp., *B. ovis* is a facultative intracellular bacterium able to survive and replicate in phagocytic and nonphagocytic cells, and establishing chronic infections in animals [6], [7]. In the absence of classical virulence factors, such as capsule and fimbriae [7], *Brucella* species require specific virulence factors for their survival, and replication in host cells [8]–[11], including the *virB*-encoded type IV secretion system (T4SS) and its secreted effector proteins. Previous studies have shown attenuation of *virB* mutant strains in either pathogenic smooth species (*Brucella melitensis, Brucella abortus,* and *Brucella suis*) or rough species (*B. ovis*) [8], [12]–[16]. *Brucella* strains lacking a functional T4SS cannot evade degradation in lysosomes and, consequently, do not reach their replicative niche in the rough endoplasmatic reticulum [17] nor establish chronic infection [9], [10], [13].

Genomic analyses of *B. ovis* resulted in the identification a pathogenicity island (BOPI-1) in chromosome II containing 28 open reading frames (ORFs), which are absent in other classical *Brucella* species [18]. This island comprises genes that potentially encode pathogenesis-associated proteins, including an ATP-binding cassette (ABC) transporter (BOV_A0504-BOV_A0500, designated *abcEDCBA*) [18], [19]. ABC transporters are responsible for nutrient uptake and the export of toxins and antibiotics, and they may play an important role in gene expression [20], [21]. In *Brucella* spp., a polysaccharide ABC transporter is required for *B. abortus* pathogenesis in the murine model [11], whereas ABC transporter proteins related to iron transport and toxin excretion were not essential for *B. abortus* chronic infection in mice [21], [22].

In *B. ovis*, a species-specific ABC transporter located at the BOPI-1 was essential for survival and replication in a mouse model and in macrophages [19]. However, it is not known what the specific role of this transporter is and whether it affects other virulence factors necessary for *B. ovis* survival in host cells. *B. ovis* is the classical *Brucella* species with lowest number of ABC transporters predicted to be functional, due to high numbers of pseudogenes in conserved *Brucella* spp. regions predicted to encode ABC systems [18], [23]. This may be one of the determinants of the low pathogenicity of *B. ovis* during animal and human infections. Hence, studying specific features of *B. ovis* may explain why it is not virulent in humans [18]. Moreover, high numbers of pseudogenes in ABC systems may allow evaluation of the pathogenic role of conserved transporters in *B. ovis* by one single gene deletion. This is less feasible in classical *Brucella* species, like *B. melitensis* and *B. abortus*, due to the presence of redundant transporters, which may compensate the function of a deleted protein.

The goal of this study was to evaluate the role of a specific ABC transporter during *B. ovis in vitro* growth, intracellular survival, and trafficking. Our results show here that the specific locus *abcEDCBA,* encoding a putative peptide importer, promotes intracellular survival by affecting T4SS protein expression at a post-transcriptional level and, consequently, contributing to *B. ovis* evasion of phagosome/lysosome fusion.

## Materials and Methods

### Bacterial strains, media and culture condition

Bacterial strains used in this study were the virulent strain *B. ovis* ATCC 25840 (WT); Δ*abcBA* mutant strain (TMS2) lacking a putative ABC transporter [19]; *B. ovis* WT and Δ*abcBA* isogenic strains expressing *mCherry* fluorescence (named TMS8 and TMS9, respectively), with the insertion of pKSoriT-*bla*-*kan*-*PsojA*-*mCherry* plasmid [24] (Table 1). All inocula were cultured on Trypticase Soy Agar (TSA, BD) plates with 5% sheep blood for three days at 37°C in 5% CO_2_, as previously described [25]. For proteomic analysis, *B. ovis* WT and Δ*abcBA* were grown in triplicate on TSA plates with 10% hemoglobin for three days. Kanamycin (Kan, 100 µg/mL) and Ampicillin (Amp, 200 µg/mL) were added to media when necessary. For strains TMS8 and TMS9, selected colonies were Amp resistant and fluorescent, as previously described [24].

**Table 1:** Bacteria and plasmids used in this study.

Bacteria/Plasmids	Description	Reference
Bacteria		
* B. ovis* WT	*B.ovis* ATCC25840	ATCC
Δ*abcBA* (TMS2)	*B.ovis*ΔBOV_A500-501:Kan^R^	[19]
* *WT-mCherry (TMS8)	*B. ovis:*pKSoriT+mCherry- Kan^R^, Amp^R^	This study
Δ*abcBA-*mCherry (TMS9)	*B. ovis ΔabcBA:*pKSoriT+mCherry- Kan^R^, Amp^R^	This study
* *STm WT	*S.*Typhimurium LT2 strain	ATCC
* *TT17573	*S.*Typhimurium LT2 opp-250(del:BC), tppB16:Tn10, dpp-101:Tn5- Kan^R^, Tet^R^	John Roth's collection
* *TMS14	TT17573:pTSabc - Kan^R^, Tet^R^, Amp^R^	This study
* E. coli* TOP10	*E. coli* for cloning	Invitrogen
* E. coli* DH5α	*E. coli* for cloning	-
Plasmids		
pKSoriT+mCherry	pKSoriT-*bla*-*kan-carb*-P*sojA*-*mCherry*	[24]
pTSabc	pBBR1-MCS4: *abcEDCBA+*6His, Amp^R^	This study

Considering that *B. ovis* does not grow adequately in conventional liquid media [26], a rich Trypticase Soy Broth (TSB, BD) was supplemented with 10% of FBS (Gibco). Strains were cultured overnight at 37°C on rotary shaker. Additionally, *B. ovis in vitro* growth was measured in TSB media supplemented with different concentrations of FBS (0, 2, 5, or 10%), nickel (NiSO_4_ at 0.5, 1 or 2 mM) or after chelation of divalent cations by adding EDTA (10, 25, or 50 mM). Strains were cultured up to 48 h at 37°C on rotary shaker.

For cloning, *E. coli* DH5α and TOP10 (Invitrogen) were grown on LB media or rich SOC media [2% tryptone, 0.5% yeast extract, 10 mM NaCl, 2.5 mM KCl, 10 mM MgCl_2_, 10 mM MgSO_4_, 20 mM glucose] and then plated on LB with selective antibiotics (Table 1).

*Salmonella enterica* serovar Typhimurium LT2 (STm WT) and the mutant STm LT2 opp-250 (del:BC), *tppB16*:Tn*10*, *dpp-101*:Tn*5* (TT17573) with non-functional peptide transporters were obtained from Dr. John Roth's strain collection, at UC Davis. Additionally, TT17573 (*oppBC tppB dpp)* strain expressing *B. ovis* ABC transporter locus (TMS14) was constructed. STm WT and mutants were grown overnight at 37°C on a rotary shaker in LB media or minimal M9 media [47.7 mM Na_2_HPO_4_, 22 mM KH_2_PO_4_, 8.6 mM NaCl, 18.6 mM NH_4_Cl, 2 mM MgSO_4_, 0.1 mM CaCl_2_] supplemented with 0.4% glucose and 1 µg/mL thiamine. Kan (100 µg/mL), tetracycline (10 µg/mL) and Amp (100 µg/mL) were added when necessary.

### Cloning and complementation

To express *B. ovis* ABC transporter locus in *S.* Typhimurium TT17573 (*oppBC tppB dpp)* strain, the entire *abcEDCBA* locus (5.6 Kb) was amplified by PCR, using genomic DNA from *B. ovis* ATCC 25840. A histidine tag (6x-his) and a stop codon were engineered into the C-terminal region (Table 2). PCR reactions were prepared with 23 µL of Supermix High Fidelity (Invitrogen), 0.7 mM of each primer abc-his (Table 2) and 2 µL of the genomic DNA. Cycling parameters, as described by [27], were as follows: denaturation at 94°C for 3 min; 35 cycles of denaturation at 94°C for 1 min, annealing at 50°C for 1 min and extension at 60°C for 6 min; and final extension at 60°C for 10 min. The 5.6 Kb product was purified from agarose gel using QIAEXII kit (Qiagen) and, then, inserted into the cloning vector pCR2.1 TOPO, following the manufacturer's instructions (Invitrogen). The insert was excised by double digestion with *Spe*I and *Kpn*I, and cloned into expression vector pBBR1-MCS4, Amp^R^ (4 Kb). To confirm the sequence and orientation of the insert, the constructed plasmid, named pTSabc, was sequenced using primers M13 (Invitrogen) (Table 2). Then, the plasmid was introduced into *S.* Typhimurium TT17573 (*oppBC tppB dpp)* by electroporation, with previous heating of the bacteria for 30 min at 50°C [28]. Colonies resistant to Tet, Kan, and Amp were selected and the ABC transporter expression in TMS14 (TT17573:*abcEDCBA*) was confirmed by Western blot, using anti-Histidine tag mouse monoclonal antibody conjugated with HRP (Lifetech).

**Table 2:** Primers for PCR used in this study.

Primer	Sequence (5′-3′)^a^	Product	Size	Reference
abc-his Fw	GGTACC TGTCCGAATAGACGGTTCGT	*B. ovis abcEDCBA* +6His	5.6 Kb	This study
abc-his Rev	ACTAGT TCAATGGTGATGGTGATGGTGAGCTTTTTCAATAACTCGAAT			
DppA Fw	ATTTTCGCCTGTGCGTTTTA	STm DppA	2.6 Kb&	This study
DppA Rev	CGGCAGTGTGATCGAAGATA			
TppB Fw	GCCCCGTTTTCACTACAAAA	STm TppB	2 Kb&	This study
TppB Rev	ATTTCGCGCAGGGTAATATG			
Opp Fw	CGATTCCGACGCTGTTTATT	STm OppBC	1.8 Kb	This study
Opp Rev	TAACGATCTTTCGGGTCGAG			
BO4 FW	TGGTATCTTCAGCCGTTCCAAG	*B. ovis abcA*	135 bp*	[19]
BO4 REV	ATCTTTGCCCGTTCCAGTCG			
M13 Fw	GTAAAACGACGGCCAG	Insert in vector	-	Invitrogen
M13 Rev	CAGGAAACAGCTATGAC			

a Underlined sequences are restriction enzymes. Bold sequence represents the histidine tag.

* Negative PCR for *B. ovis* mutant strain.

& Negative PCR for TT17573 strain.

The deletion of *oppBC* and transposon insertion into *dppA* and *tppB* in *S.* Typhimurium TT17573 were confirmed by PCR as described, using pairs of primers shown in Table 2.

### S. Typhimurium lethality assay

Considering that STm has three types of peptide ABC transporters, which mainly transport dipeptides (Dpp), tripeptides (Tpp) or oligopeptides (Opp) [29]–[31], we attempted to use this organism to predict the function of *B. ovis* transporter by evaluating bacterial resistance to toxic peptides [29], [32].

For the lethality assay, STm WT, STm TT17573 (*oppBC tppB dpp*), and TMS14 (TT17573:*abcEDCBA*) mutant expressing the *B. ovis abcE-A* transporter were grown overnight in M9 liquid media. Each bacterial strain was adjusted to 3×10^5^ CFU/mL in fresh M9 with 0.7% noble agar (BD) and layered over M9 agar plates containing antibiotics. After solidifying, 7 mm-filter paper disks containing 0.5 mg and 1 mg of alafosfalin (L-Alanyl-L-1-aminoethylphosphonic acid, Sigma-Aldrich) or 0.2 mg and 0.4 mg of trilysine (Sigma-Aldrich) were placed onto the plate. After drying the disks, plates were incubated for 16 h at 37°C. The ability of a toxic peptide to inhibit bacterial growth was quantified by determining the diameter (mm) of the inhibitory zone surrounding a filter paper disk with the toxic peptides. Assays were performed three times independently, with triplicate samples.

### Proteomic analysis by Differential Gel Electrophoresis (DIGE)

Protein expression of WT and Δ*abcBA B. ovis* were compared by DIGE during *in vitro* growth in rich media. For each bacterial strain, triplicates grown independently were used. Protein was extracted with 2 vol of lysis buffer (8 M urea, 2 M thiourea, 4% w/v CHAPS, 40 mM Tris1M, mix of protease inhibitors) (GE Healthcare), followed by 3 h of agitation and cellular lysis by passing through a 26G needle. Lysates were centrifuged at 20,000×g for 30 min at room temperature, and the supernatants were recovered, quantified by 2D Quant Kit (GE Healthcare), and kept at −80°C. A pool of protein extracts obtained from triplicate samples was used.

To identify differentially expressed proteins between *B. ovis* WT and Δ*abcB* mutant, the protein mixture (50 µg) of each strain was labeled with 400 pmol of either Cy3 or Cy5 dye (GE Healthcare), according to the manufacturer's instructions. A protein mixture of both strains (50 µg) was labeled with Cy2 dye as internal control. These reactions were carried out on ice for 30 min in the dark and quenched with 1 µL of lysine (10 mM) for 10 min on ice. Four DIGE gels were done and a dye-swap was performed. All labeled proteins (150 µg) were added to 3.4 µL of immobilized pH gradient (IPG) buffer (10 µL/mL), 450 µg of unlabeled protein, and IEF buffer (8 M urea, 2 M thiourea, 4% CHAPS, 0.0025% bromophenol blue, 10 mg/mL dithiothreitol) in a total volume of 340 µL per IPG strip (18 cm, pH 4–7, GE Healthcare). The samples were incubated overnight with the IPG strips, submitted to isoelectric focusing using Ettan IPGphor system (GE Healthcare), followed by incubation in equilibrium solution (50 mM Tris-HCl, pH 8.8, 6 M urea, 30% glycerol, 2% SDS, 0.002% bromophenol blue, and 125 mM DTT) for 15 min and an additional incubation in a new solution containing 13.5 mM iodoacetamide instead of DTT.

Electrophoresis was performed in 12% SDS-PAGE using an Ettan Electrophoresis unit (GE Healthcare) at 10 mA/gel for 1 h, followed by 45 mA/gel until the dye front reached the bottom of the gel. Each gel was scanned using Typhoon FLA 9000 (GE Healthcare) with excitation/emission wavelengths of 488/520, 532/580, and 633/670 nm for Cy2, Cy3, and Cy5 dyes, respectively. Gel images were analyzed using DeCyder 2D software, Version 7.0 (GE Healthcare). Spots with p-value <0.05 and average of volume ratio over 1.5 were selected for mass spectrometry (MS) identification. To extract the spots of interest, all DIGE gels were subsequently stained with colloidal Coomassie Brilliant blue G-250, as previously described [33].

### Mass spectrometry (MS) and prediction of protein interactions

Differentially expressed spots between WT and Δ*abcBA B. ovis* were excised from gel, treated with trypsin, and desalted using Zip-Tips (Millipore Corporation), as described elsewhere [34]. Each sample was mixed with 0.5 vol of saturated matrix solution (10 mg/mL α-cyano-4-hydroxycinnamic acid in 50% acetonitrile/0.1% trifluoroacetic acid). Then, samples were spotted on MTP AnchorChip 600/384 (Bruker Daltonics) and let it dry at room temperature. For protein identification, raw data were acquired on a MALDI-TOF/TOF AutoFlex III instrument (Bruker Daltonics) in the positive/reflector mode controlled by FlexControl software. Instrument calibration was previously done using peptide calibration standard II (Bruker Daltonics) as a reference.

Data from mass spectrometry were aligned against all non-redundant protein sequence database from NCBI (http://www.ncbi.nlm.nih.gov) using the MASCOT software MS/MS ion search tool (http://www.matrixscience.com). The parameters in this search were as follows: no restriction on protein molecular weight, loss of a trypsin cleavage site, variable modifications of methionine (oxidation), cysteine (carbamidomethylation), and pyroglutamate formation at the N-terminal glutamine. The mass tolerance for searched peptides were 0.8 Da for MS spectra and 0.6 Da for MS/MS spectra. Peptides were identified when the scoring value exceeded the identity or extensive homology threshold value calculated by the MASCOT (p<0.05).

Interactions of identified proteins were predicted by using the STRING software (http://string-db.org), as previously described [35]. The evidence mode was set up at an average confidence level 0.4 and the search parameters included: neighborhood, gene fusion, co-occurrence, co-expression, experiments, databases, and text mining.

### HeLa cell culture and infection

HeLa cells were cultured in a 75 cm^2^ flasks with Dulbecco's modified Eagle medium (DMEM, Gibco) supplemented with 10% FBS, at 37°C with 5% of CO_2_. When reaching 80–90% of confluence, cells were treated with Trypsin-EDTA 0.05% for 10 min, and seeded overnight in a 24-well plate at a density of 1×10^5^ cells per well. The next day, HeLa cells were infected as previously described [36], with some modifications for *B. ovis*. Briefly, *B. ovis* WT, Δ*abcBA* and *mCherry-*expressing isogenic strains (TMS8 and TMS9) were grown for 3 days, resuspended in DMEM supplemented media, and added 0.5 mL to each well, to infect HeLa cells with the multiplicity of infection (MOI) of 1000. The plates were centrifuged at 400 × *g* for 5 min at room temperature and incubated for 30 min at 37°C in 5% CO_2_. The cells were washed three times with Dulbecco phosphate-buffered saline (DPBS) to remove free bacteria, followed by the addition of 0.5 mL of DMEM supplemented media with 50 mg/mL of gentamicin into the wells, to kill the extracellular bacteria. This was considered the zero time point.

In order to determine bacterial survival, the medium was aspirated at 0, 8, 24, and 48 h after infection and HeLa cells were lysed with 0.5 ml of 0.5% Tween 20, followed by rising each well with 0.5 mL of PBS. Viable bacteria were quantified by serial 10-fold dilutions in sterile PBS and plating on TSA with 5% sheep blood. This experiment was performed in triplicate and repeated three times.

### Brucella ovis confocal microscopy

HeLa cells were seeded overnight on a 12-mm glass coverslips in a 24-well plate at a density of 3×10^4^ cells per well. The cells were infected with *B. ovis* mCherry-expressing WT or Δ*abcBA* strain with MOI of 1000. At 8, 24, and 48 h post infection, each coverslip was washed three times with PBS, fixed with 3% paraformaldehyde (pH 7.4) at 37°C for 20 min, followed by three washes with PBS and incubation in 50 mm NH_4_Cl in PBS for at least 10 min, to quench free aldehyde groups. Samples were permeabilized with 10% horse serum and 0.1% saponin in PBS for 30 min at room temperature. After removing the coverslips, each sample was labeled with rabbit anti-human LAMP-1 antibody (Thermo Scientific), by inverting the coverslips onto droplets of primary antibody diluted (1∶1000) in 10% horse serum and 0.1% saponin solution in PBS. After incubating for 1 h at room temperature, each sample was washed with PBS and labeled with diluted (1∶1000) Alexa Fluor 488 anti-rabbit antibody (Lifetech) for 1 h at room temperature. Then, cells were washed twice with 0.1% saponin in PBS, once in PBS, once in distilled H_2_O and mounted in Mowiol 4–88 mounting medium (Calbiochem). Samples were analyzed on a Carl Zeiss LSM 510 confocal laser scanning microscope for image acquisition. Confocal images of 1024×1024 pixels were acquired as projections of three consecutive slices with a 0.38-μm step and gathered using Adobe Photoshop CS5. To quantify *B. ovis* infection in cells and LAMP-1^+^ compartment colocalization, at least 100 bacteria and 100 cells per sample were counted. All experiments were performed independently three times and in triplicate.

### Expression of T4SS proteins by Western blot

The expression of VirB proteins, which constitute the T4SS, were evaluated during *in vitro* growth of *B. ovis* WT and Δ*abcBA* strains. Each sample was cultured on TSA plates with 5% of sheep blood for three days and subsequently transferred to TSB with 10% FBS, at a starting OD_600_ of 0.1. After incubating overnight on a rotary shaker at 37°C, cells from 1 mL of each culture were pelleted by centrifugation and resuspended in SDS-PAGE buffer. VirB expression was also analyzed after growth of *B. ovis* on TSA plates containing 5% of sheep blood, by scraping an aliquot straight from the plate and resuspending in SDS buffer.

Considering that T4SS expression in classical *Brucella* species is induced by acidic pH and nutrient-poor conditions [37], expression of VirB proteins under these conditions was assayed. To this end, 10^9^ CFU of each strain was suspended in modified minimal E medium pH 5.0, and incubated for additional 6 h at 37°C on a rotary shaker, as previously described [38]. VirB protein expression is also promoted by urocanic acid, which induces expression of two T4SS regulators (HutC and VjbR) in an acidic environment [39]. Therefore, *B. ovis* WT and Δ*abcBA* (10^9^ CFU) were suspended in modified minimal E medium (pH 5.0) supplemented with urocanic acid 5 mM or glutamic acid 5 mM (control), and incubated for additional 4 h at 37°C on a rotary shaker. Assays were performed at least three times independently for each strain.

For Western blot, bacterial proteins were extracted by heating the samples for 10 min in 4% SDS buffer. The total protein (10 to 20 µg) was electrophoresed on a 12% SDS-PAGE gel, and transferred to a nitrocellulose membrane. Membranes were blocked in blocking solution (PBS containing 2% non-fat skim milk powder and 0.05% Tween 20) for 1 h and incubated for 1 h with rabbit anti-VirB8, anti-VirB9 or anti-VirB11 polyclonal antibody diluted in blocking solution (1∶5000). Then, membranes were washed three times with blocking solution and incubated for another 1 h with diluted (1∶5000) goat anti-rabbit IgG antibody (Biorad) conjugated with horseradish peroxidase (HRP). HRP activity was detected with a chemiluminescent substrate (Perkin-Elmer). As a loading control, same sample concentrations used in Western blot were loaded on a separate SDS-PAGE gel and stained with Coomassie Brilliant Blue.

### Bacterial RNA extraction and Real Time RT-PCR

To compare gene expression of *B. ovis* WT and Δ*abcBA* strains during *in vitro* growth, RNA was extracted from 1 mL of bacterial samples grown on TSB media with 10% FBS for 24 h at 37°C on rotary shaker. RNA extraction was carried out using TRI reagent (Molecular Research Center, Cincinnati) as previously described [13], followed by RNA purification with RNeasy Minelute cleanup kit (QIAGEN) and DNase treatment (Invitrogen) for 1 h at 37°C. Real-time PCR was performed using TaqMan reverse transcription reagent (Applied Biosystems) with 10 µL of RNA from each sample in a 60((L volume. To assess whether there was genomic DNA contamination in samples, a new 30((L mix volume of TaqMan reagent was performed with 5((L of RNA, without adding reverse transcriptase enzyme. Four (L of cDNA was used as the template for each reverse transcription-PCR (RT-PCR) in a 25 µL volume, with 12.5 µL of SYBR Green (Applied Biosystems) and 0.3 µM of each primer listed in Table 3. Data were analyzed using the comparative Ct method (Applied Biosystems). Transcript levels of *virB1*, *virB8*, *vjbR, hutC, abcA,* and *abcC* were normalized with mRNA levels of the housekeeping 16S ribosomal gene (Table 3). Ct values of *B. ovis* WT genes were expressed in relation to *B. ovis* Δ*abcBA* strain.

**Table 3:** Primers for *Brucella ovis* Real Time RT-PCR.

Primer		Sequence	Reference
Univ RT	Fw	ACTCCTACGGGAGGCAGCAGT	This study
	Rev	ATTACCGCGGCTGCTGGC	
BOVA500 RT	Fw	CTACGCTCGCGCTCTCTATT	This study
	Rev	ACCGCCAGCGACATATAAAC	
BOVA503 RT	Fw	ATGTGGCCTACGCTGAAACT	This study
	Rev	AGCCAGAATTGCGGTAGAGA	
VirB1 RT	Fw	TGTTACTACGCCGGCAACTT	This study
	Rev	CAGCAATCGGCTTTGTGGTC	
VirB8 RT	Fw	TAAAGAACGGGCAGGGCAAT	This study
	Rev	CACGGTAATGGTGCCGAAAG	
VjbR RT	Fw	GGTTTTTCAGGAAGACGCTC	This study
	Rev	AAGATTTCCCAGGCCGTGC	
HutC RT	Fw	TTTGAACACGAGCTGACCGA	[36]
	Rev	TGCGATTGCGGGAACGACA	

### Statistical analyses

All CFU data were logarithmically transformed and submitted to analysis of variance (ANOVA). For confocal microscopy, all percentage data were submitted to angular transformation prior ANOVA. Means of groups were compared with Tukey's test (GraphPad InStat 3) and considered significant when p<0.05. Confocal microscopy and real time data represent geometric mean and standard error of three independent experiments. For real time PCR, Ct values were compared between groups by Student T test, and considered significant when p<0.05.

## Results

### Prediction of ABC transporter function during in vitro growth

According to genomic analysis of *B. ovis* [18], [23], the *B. ovis abcEDCBA* locus was previously predicted to encode a peptide importer [19]. Additional analysis was performed in this study, by aligning the nucleotide sequence to sequences available at the NCBI protein database (BLASTx).

Two proteins encoded by *abcA* (BOV_A0500) and *abcB* (BOV_A0501), which were deleted in the *abcBA* mutant strain, are predicted to be ATPases of ABC systems, with conserved Walker A and B motifs. Therefore, deletion of *abcA* and *abcB* would lead to inactivation of the transporter. Both ATPases showed 99% identity only with *Brucella pinnipedialis* B2/94 and *Brucella* sp. 63/311, and 91% identity with two phylogenetically related bacteria (*Ochrobactrum anthropi* and *Ochrobactrum intermedium*). Proteins encoded by *abcE-C* (BOV_A0504-502) were identical to a conserved group of ABC systems functioning in uptake of dipeptides, oligopeptides and nickel (Dpp/Opp/Nik). Both *abcD* and *abcC* are predicted to be transmembrane proteins, whereas *abcE* encodes a predicted substrate-binding protein, suggesting a function of the ABC system in substrate uptake.

The role of the *B. ovis abcEDCBA* transporter was evaluated during *in vitro* growth, using a liquid medium that allowed for exponential growth of both WT and Δ*abcBA* strains. Both strains showed limited growth after 24 h in TSB media (Figure S1A), which is a standard laboratory media for *Brucella* spp. [40]. When TSB was supplemented with different concentrations of FBS (2, 5, or 10%), WT and Δ*abcBA* showed equally proportional growth, reaching maximal growth with 10% FBS (Figure S1A). For further *B. ovis* experiments, TSB with 10% FBS was used as the standard liquid media.

Considering the genomic prediction of *B. ovis abcEDCBA-*encoded proteins as a nickel transporter (Nik), it was evaluated if *in vitro* growth was impacted by adding or removing nickel from the growth medium. Addition of NiSO_4_ (0.5, 1, or 2 mM) to TSB with 10% FBS did not increase growth of either *B. ovis* Δ*abcBA* mutant, or the WT strain after 48 h (Figure S1B). Also, chelation of divalent cations, including nickel, by adding EDTA at 10, 25 or 50 mM into the media equally limited the growth of both strains. These results show that deletion of the *abcEDCBA*–encoded ABC transporter did not restrict nickel uptake or *in vitro* growth of Δ*abcBA B. ovis.*

### B. ovis abcEDCBA does not complement a peptide transport-deficient strain of S. Typhimurium

To assess the predicted function of *B. ovis* ABC transporter as a peptide importer, this protein was constitutively expressed in *S.* Tm TT17573, which carries spontaneous mutations in the genes *oppBC tppB dpp*, resulting in non-functional peptide transporters for oligopeptides (Opp), tripeptides (Tpp) and dipeptides (Dpp). Previous studies have characterized peptides that are toxic for STm when taken up by a specific type of transporter [29], [32]. Due to easy growth of STm in protein-restricted media, sensitivity of STm WT, STm TT17573 (*oppBC tppB dpp*), and TT17573 strain complemented with *abcEDCBA* (TMS14) were evaluated against trilysine and alafosfalin, which are imported by Tpp and Opp transporters, respectively (Figure S2). For detection of *abcEDCBA* expression in *S.* Typhimurium, a 6x-His tag was engineered into the C-terminus of AbcA.

STm WT and TT17573 (*oppBC tppB dpp*) demonstrated similar growth and metabolic activity by tetrazolium reduction in minimal M9 media (Figure S2). STm WT was susceptible to trilysine and alasfosfalin at two different concentrations (Figure S3A-B). However, STm TT17573 (*oppBC tppB dpp*) and TMS14 (TT17573:*abcEDCBA*) were resistant to toxic peptides (Figure S3A), suggesting that these strains have non-functional Tpp and Opp transporters. Introduction of *B. ovis abcEDCBA* into STm TT17573 did not confer peptide uptake, although expression of *abcA* was confirmed by Western blot (Figure S3C). These results suggested that *B. ovis abcEDCBA* does not function in *S*Tm as an oligopeptide or tripeptide importer.

### Inactivation of Brucella ovis abcEDCBA affects the abundance of metabolic and virulence-associated proteins during in vitro growth

**Figure 1:**
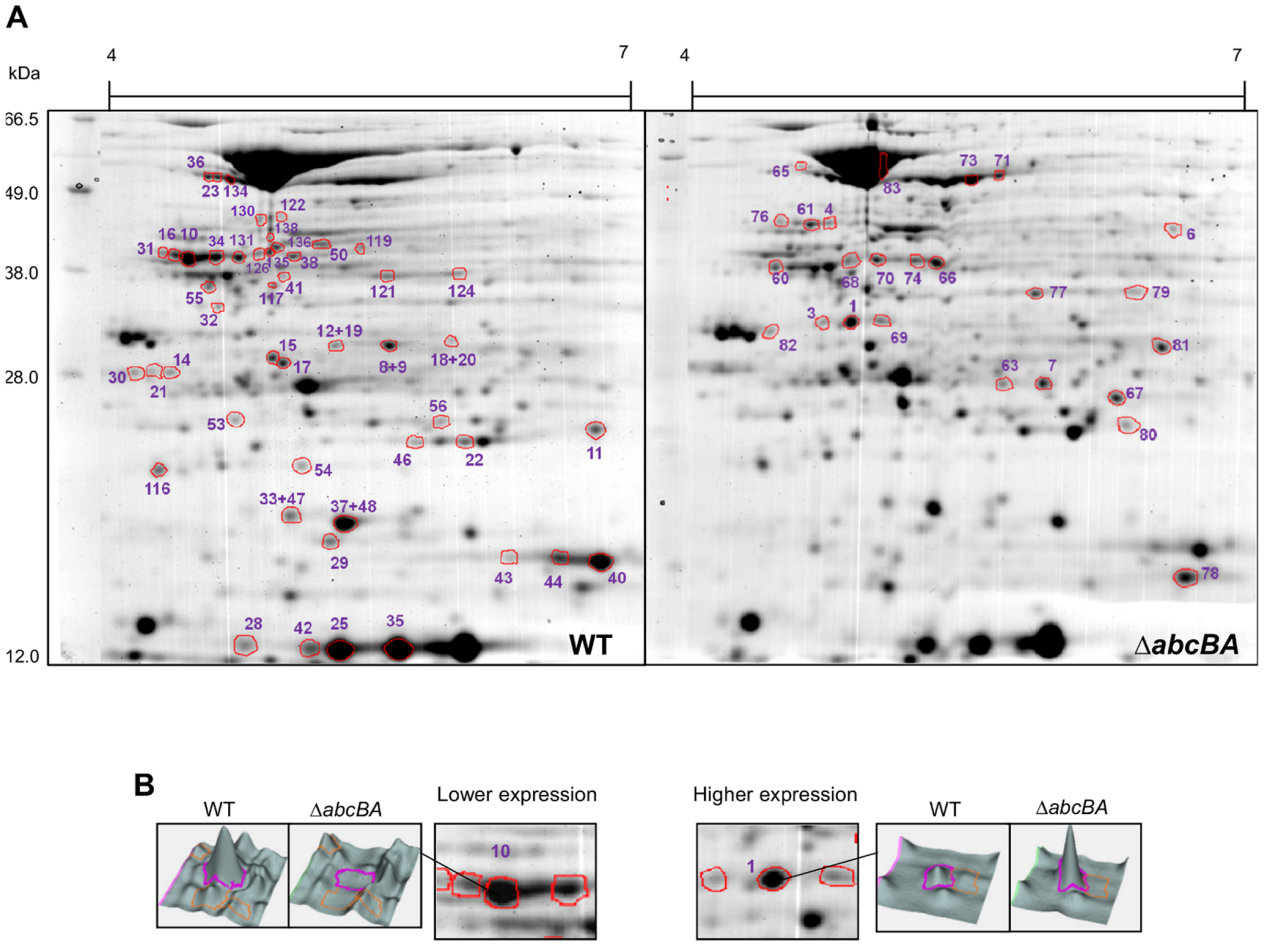
Representative images of bi-dimensional DIGE of protein extracts from *Brucella ovis* wild type and Δ*abcBA* mutant. (A) Enumerated spots were differently expressed (p<0.05) between Δ*abcBA* and WT strains and selected for mass spectrometry. Spots with lower expression were excised from *B. ovis* WT gel (left panel), whereas spots with higher expression were excised from *B. ovis* Δ*abcBA* gel (right panel). (B) Topographic 3D images of the signal intensity correspond to a lowly expressed (left) and a highly expressed (right) spot.

To gain insight into the role of *abcEDCBA* in the biology of *B. ovis,* differential expression of proteins between WT and Δ*abcBA B. ovis* strains was evaluated by 2D-DIGE. A representative image of the protein profile for each strain is shown in Figure 1. By DeCyder 2D image analysis software (GE Healthcare). Considering a volume ratio higher than 1.5; 100 spots had differential expression between the strains (Figure S4), whereas 78 spots were visualized in the gel and extracted for mass spectrometry (MS) identification (Figure 1A). Among these, 55 spots were successfully identified by MS/MS, whereas 40 spots (72,7%) had lower expression (Tables 4 and S1) and 15 spots had higher expression (Tables 5 and S2) in Δ*abcBA B. ovis*. Tables S1 and S2 show MS data for each spot, including peptide sequence, score, percentage of coverage, predicted and experimental values of isoelectric point (pI) and molecular weight (MW).

**Table 4:** Functional classification of proteins with lower expression in Δ*abcBA Brucella ovis* (p<0.05) during in vitro growth in rich neutral media.

Spot^a^	Protein ID^b^	Volume Ratio	Acession number^c^
	**Outer membrane proteins**		
8+9	31 kDa immunogenic protein	−27.37	gi 5202891
12+19	31 kDa immunogenic protein	−6.08	gi 5202891
18+20	31 kDa immunogenic protein	−4.84	gi 5202891
	**Sugar ABC transporters**		
10	ABC transporter sugar binding protein	−14.1	gi 1198362*
16	ABC transporter sugar binding protein	−6.48	gi 1198362*
31	ABC transporter sugar binding protein	−3.56	gi 1198362*
34	ABC transporter sugar binding protein	−3.36	gi 1198362*
14	D-ribose-binding periplasmic protein precursor	−7.08	gi 1198207*
21	D-ribose-binding periplasmic protein precursor	−4.3	gi 1198207*
30	D-ribose-binding periplasmic protein precursor	−3.75	gi 1198207*
32	D-xylose-binding periplasmic protein precursor	−3.44	gi 1197917*
50	ABC transporter periplasmic glycerol-3-phosphate-binding prot	−2.32	gi 5204123
117	ABC transporter periplasmic sugar-binding protein	−2.25	gi 5204064
	**Aminoacid ABC transporters**		
15	glycine betaine/L-proline ABC transporter binding protein prox	−7.08	gi 1198322*
23	oligopeptide ABC transporter substrate-binding protein	−4.14	gi 5203758
36	oligopeptide ABC transporter substrate-binding protein	−3.23	gi 5203758
134	oligopeptide ABC transporter substrate-binding protein	−1.53	gi 5203758
38	ABC transporter periplasmic amino acid-binding protein	−2.94	gi 5204134
55	ABC transporter periplasmic substrate-binding protein	−2.08	gi 5202558
136	ABC transporter periplasmic amino acid binding protein	−1.52	gi 5203023
	**Protein folding and stress proteins**		
25	co-chaperonin GroES	−4.08	gi 5203793
42	co-chaperonin GroES	−2.67	gi 5203793
35	Acid stress chaperone HdeA	−3.23	gi 5203776
33+47	DNA starvation/stationary phase protection protein Dps	−2.94	gi 5201095
37+48	DNA starvation/stationary phase protection protein Dps	−2.74	gi 5201095
40	superoxide dismutase, Cu-Zn	−2.83	gi 5203847
44	superoxide dismutase, Cu-Zn	−2.62	gi 5203847
22	superoxide dismutase, Fe-Mn family	−4.17	gi 5202836
46	superoxide dismutase, Fe-Mn family	−2.59	gi 5202836
131	superoxide dismutase, Fe-Mn family	−1.57	gi 5202836
	**Metabolic enzymes**		
	*Nucleotide metabolism*		
29	nucleoside diphosphate kinase	−3.79	gi 5201998
	*Protein metabolism*		
50	isovaleryl-CoA dehydrogenase	−2.32	gi 5202101
	*Sugar metabolism*		
56	putative translaldolase	−2.05	gi 5201313
	**Protein biosynthesis**		
11	ribosome recycling factor	−9.56	gi 5202626
	**Vitamin biosynthesis**		
53	riboflavin synthase subunit alpha	−2.14	gi 5202369
	**Unknown function**		
17	hypothetical protein	−5.56	gi 5202567

a The numbers correspond to specific spots as indicated in Figure 5.

b Predicted function of proteins according to NCBI.

c Accession numbers correspond to *B. ovis* protein database in NCBI.

*Pseudogenes in *B. ovis* with corresponding accession number of *B. melitensis* 16M protein database.

**Table 5:** Functional classification of proteins with greater expression in Δ*abcBA Brucella ovis* (p<0.05) during in vitro growth in rich neutral media.

Spot^a^	Protein ID^b^	Volume Ratio	Acession number^c^
	**ABC transporters**		
1	ABC transporter periplasmic amino acid-binding protein	3.66	gi 5201724
3	ABC transporter periplasmic amino acid-binding protein	2.33	gi 5201724
69	ABC transporter periplasmic amino acid-binding protein	1.76	gi 5201724
71	nickel ABC transporter substrate binding protein	1.69	gi 5204178
73	nickel ABC transporter substrate binding protein	1.67	gi 5204178
	**Sugar metabolism**		
4	succinyl-CoA synthetase beta chain	2.32	gi 5201923
61	succinyl-CoA synthetase beta chain	1.95	gi 5201923
66	malate dehydrogenase	1.79	gi 5201532
70	malate dehydrogenase	1.7	gi 5201532
	**Protein metabolism**		
76	zinc protease	1.54	gi 5203839
	**Transcriptional regulation**		
67	NAD(P)H dehydrogenase (quinone)	1.78	gi 5201943
	**Protein folding**		
83	60kDa chaperonin GroEL	1.5	gi 5203035
	**Specific molecular function**		
7	metal-dependent hydrolase	2.04	gi 5203223
63	metal-dependent hydrolase	1.85	gi 5203223
81	aldo/keto reductase family, oxidoreductase	1.52	gi 5203362

a The numbers correspond to specific spots as indicated in Figure 5.

b Predicted function of proteins according to NCBI.

c Accession numbers correspond to *B. ovis* protein database in NCBI.

A total of 22 proteins had lower expression in *B. ovis* Δ*abcBA*, which included the following functional groups: outer membrane protein (Omp31); predicted amino acid and sugar ABC transporter binding proteins (ribose, glycerol, xylose, oligopeptide, and glycine binding proteins); protein folding (chaperonin GroES, acid stress chaperone HdeA); stress proteins (DNA starvation protein HdeA, superoxide dismutase Cu/Zn and Fe-Mn); metabolic enzymes (nucleoside diphosphate kinase); and protein and vitamin biosynthesis (Table 4).

Four proteins, which corresponded to nine spots, were not identified in *B. ovis* protein database from NCBI. However, the peptide sequences were identified as periplasmic proteins of sugar and aminoacid ABC transporters in other classical *Brucella* spp. species. Therefore, for these spots, the *B. melitensis* 16M database was used to predict protein function and interaction. Interestingly, by aligning the nucleotide sequence of protein-encoded genes in *B. melitensis* with *B. ovis* database (BLASTn), all four genes were identified as pseudogenes in *B. ovis*. These findings reveal the expression of four distinct ABC systems in *B. ovis* during in vitro growth, which were previously annotated incorrectly as pseudogenes.

Nine proteins had higher expression levels in Δ*abcBA B. ovis*, including: ABC transporters (nickel NikA and amino acid binding proteins); sugar metabolism (succinyl-CoA synthetase and malate dehydrogenase); protein metabolism (zinc protease); and transcriptional regulation (dehydrogenase quinone) (Table 5).

Additionally, to detect virulence and metabolic differences between WT and Δ*abcBA* strains, interaction network predictions were performed among identified proteins with lower and higher expression in Δ*abcBA B. ovis* (Figures S5 and S6). Downregulated proteins interacted mainly with two protein groups related to metabolic stress response and ABC transporters (Figures S5), whereas upregulated proteins interacted with a major protein group related to carbohydrate metabolism (Figures S6). Taken together, these results show that *B. ovis* specific ABC peptide transporter plays an important role in metabolism and expression of virulence proteins of *B. ovis*.

### B. ovis ΔabcBA is not able to survive intracellularly in HeLa cells

A previous study showed that *B. ovis* Δ*abcBA* did not survive intracellularly in murine macrophages and was attenuated early during infection in a mouse model [19]. However, we were interested in determining whether the inability of the Δ*abcBA* mutant to survive intracellularly reflected increased susceptibility to macrophage-specific bactericidal effects [36], [41], or whether it had a general defect in intracellular survival. Since epithelial cell lines can allow the characterization of infection and trafficking of extremely attenuated *Brucella* spp. strains [36], [41], HeLa cells were infected with *B. ovis* WT or Δ*abcBA* strain at MOI 1∶1000 and infection was evaluated at 0, 8, 24, and 48 hours post infection (hpi).

**Figure 2:**
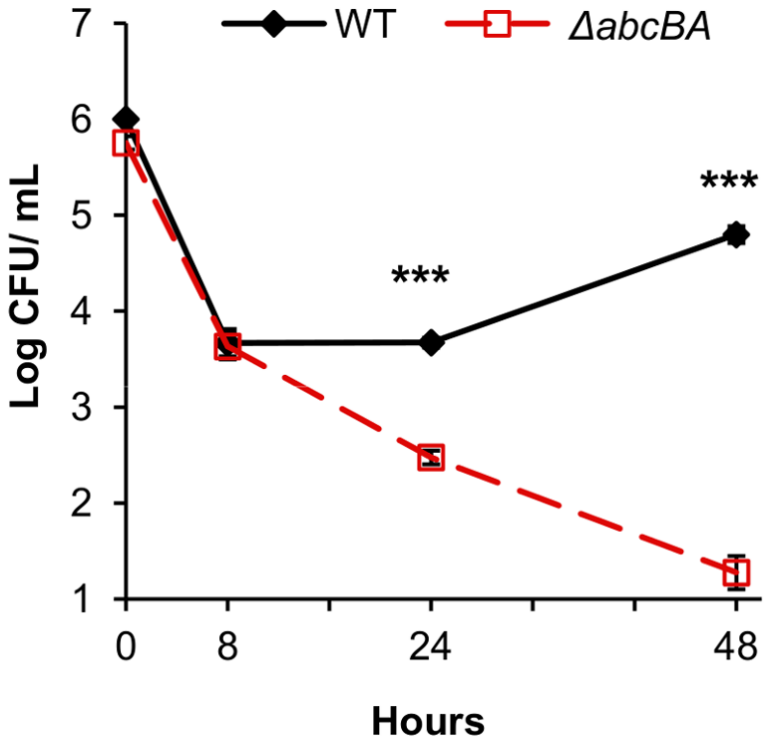
Intracellular survival of wild type or Δ*abcBA Brucella ovis* mutant in HeLa cells. HeLa cells infected with MOI 1:1000 of WT or Δ*abcBA of B. ovis* and intracellular infection measured at 0, 8, 24, and 48 hours post infection (hpi). Data represent geometric mean and standard error of three independent experiments (***p<0.001).

*B. ovis* WT and Δ*abcBA* strains demonstrated similar internalization at zero time point and kinetics of infection up to 8 µhpi in HeLa cells. At 24 µhpi, intracellular CFU numbers of the Δ*abcBA* mutant strain decreased significantly compared to the WT strain (p<0.001). *B. ovis* Δ*abcBA* infection was controlled until 48 µhpi, whereas WT *B. ovis* was able to survive and replicate in HeLa cells (Figure 2). Overall, the predicted ABC importer is required for *B. ovis* intracellular survival in human epithelial cell line and, consequently, for establishing persistent *in vitro* infection. Conversely, the results suggest that this transporter is not necessary for *B. ovis* internalization or early infection, although it may have a crucial role for the bacteria to reach the replicative niche at later time points.

Moreover, the kinetics of infection of *B. ovis* WT and Δ*abcBA* isogenic strains constitutively expressing mCherry (named TMS8 and TMS9) were evaluated in HeLa cells, to confirm that the fluorescent protein expression did not interfere with their phenotype. Both WT*-*mCherry and Δ*abcBA-*mCherry strains exhibited identical infection as shown in Figure 2, which allowed us to study *B. ovis* intracellular trafficking by confocal microscopy.

### ABC transporter enables Brucella ovis to evade the phagolysosome fusion

**Figure 3:**
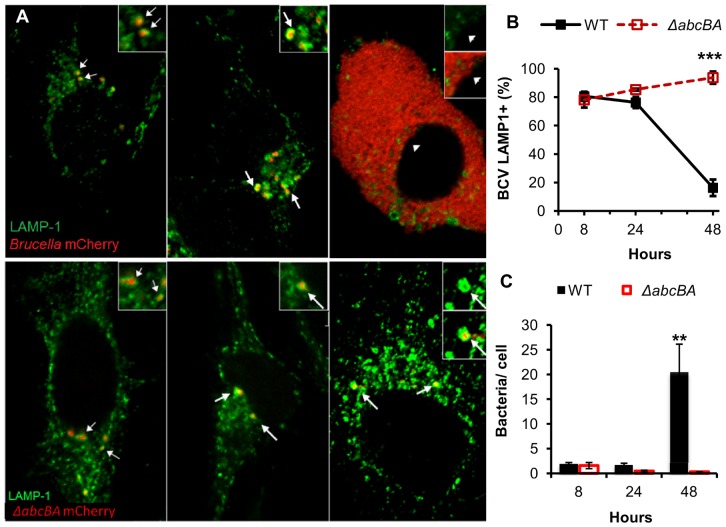
Intracellular trafficking of *Brucella ovis* in HeLa cells by confocal microscopy. (A) Intracellular trafficking of WT *B. ovis* (upper panel) and Δ*abcBA* mutant (lower panel) expressing mCherry (red) during HeLa cell infection. LAMP-1^+^ compartment is labeled in green. (B) Average percent of colocalized *Brucella* containing vacuole (BCV) and LAMP-1^+^ during WT *B. ovis* (black column) and Δ*abcBA* (red column) infection. (C) Average number of bacteria per cell during the course of infection. Data represent mean and standard deviation of at least 100 cells from three independent experiments (**p<0.01; ***p<0.001).

To understand the role of *abcEDCBA* in intracellular survival of *B. ovis*, WT *B. ovis* intracellular trafficking was characterized in HeLa cells by confocal microscopy and compared to the Δ*abcBA* strain at 8, 24, and 48 µhpi. As illustrated in Figure 3, WT-mCherry and Δ*abcBA-*mCherry strains had similar patterns of colocalization with LAMP-1 in HeLa cells at 8 and 24 µhpi, when most of *Brucella*-containing vacuole (BCV) colocalized with LAMP-1^+^ compartment, as shown in green. At 48 µhpi, higher numbers of mCherry-*B. ovis* were seen in HeLa cells infected with WT, of which approximately 80% were able to exclude LAMP-1 from their BCV (Figure 3A-C). Conversely, significantly lower numbers of Δ*abcBA-*mCherry were seen in HeLa cells at 48 µhpi and more than 90% of the bacteria colocalized with LAMP-1 (Figure 3B-C). These results reveal that WT *B. ovis* is able to avoid phagosome/lysosome fusion and to replicate, whereas the Δ*abcBA* mutant remained within lysosomes, explaining its inability to survive in a human epithelial cell line. Therefore, the ABC transporter is necessary for *B. ovis* intracellular survival and replication at later stages of infection, potentially by promoting exclusion of lysosomal markers.

### Lack of the Brucella ovis abcBACDE-encoded transporter reduces the levels of VirB proteins

**Figure 4:**
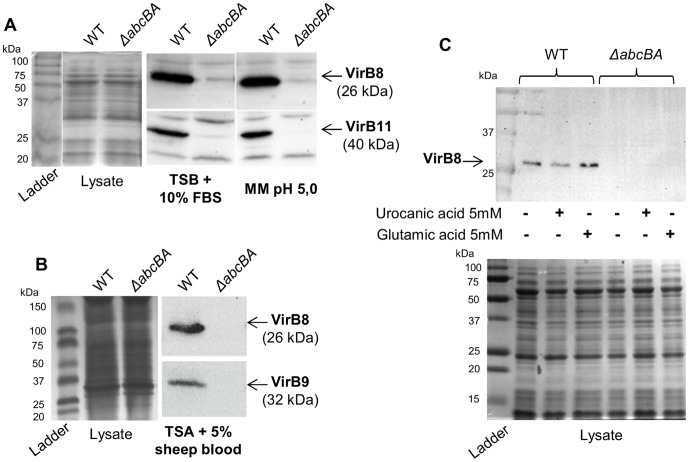
ABC transporter dependent expression of *virB*-encoded proteins in *Brucella ovis*. Western blot analysis of WT and Δ*abcBA B. ovis* strains for VirB8, VirB9, and VirB11 expression in different growth conditions. (A) Bacterial strains grown in liquid rich media (middle column) or transferred to minimal media (MM) at pH 5.0 (right column). (B) Samples taken from TSA plate with 5% sheep blood at 3-day growth (right column). Left columns show equal protein concentration of lysate. (C) WT and Δ*abcBA* strains grown for additional 4 h on MM pH 5.0 supplemented with 5 mM of urocanic acid or glutamic acid (upper panel). Lower panel shows equal protein concentration of lysate. Figures are representative of three independent experiments.

The T4SS is one of the main virulence mechanisms for *Brucella* spp., and it is required for intracellular survival and replication by promoting exclusion of lysosomal proteins from the BCV [17], [41]. Taking into account that Δ*abcBA* mutant lost the ability to exclude LAMP1, we analyzed expression of T4SS components by *B. ovis* WT and Δ*abcBA* during *in vitro* growth in rich and nutrient-limited media, using Western blotting (Figure 4).

Both strains were grown on rich liquid media (TSB with 10% FBS) and, then, transferred to modified minimal E media (MM) at pH 5.0, which promotes *in vitro* expression of the T4SS in *Brucella* spp. [37], [42]. Interestingly, unlike what was described for other *Brucella* spp., WT *B. ovis* expressed VirB8 (26 kDa) and VirB11 (40 kDa) in both neutral and acidic media. Conversely, the Δ*abcBA* mutant had weak expression of VirB proteins, including in the minimal acid media, when compared to WT *B. ovis* (Figure 4A). Expression of VirB8 and VirB9 (32 kDa) were also evaluated after standard 3-day growth on TSA plate with 5% sheep blood; however, only WT *B. ovis* was able to express VirB proteins in this condition (Figure 4B). Since *virB* expression in *Brucella* spp. has been shown to be induced poor nutritional environment with low pH [38], [39], [43], [44], we evaluated *virB* expression by *B. ovis* and *B. abortus* cultured in vitro in rich neutral medium. Indeed, *B. ovis* expressed both VirB8 and VirB11 under these conditions, whereas *B. abortus* did not express these two proteins encoded by the *virB* operon (Figures S7).

Previous studies have shown that adding urocanic acid into minimal acid media induces *in vitro* transcription of T4SS in *Brucella* sp., by increasing the expression of two *virB*-regulatory proteins, HutC and VjbR [39], [43]. To determine whether expression of *virB* genes was affected upstream of HutC and VjbR, VirB8 expression was analyzed after transferring WT and Δ*abcBA B. ovis* into MM media pH 5.0 supplemented with 5 mM of urocanic acid or 5 mM of glutamic acid (control). As illustrated in Figure 4C, even in the presence of urocanic acid, Δ*abcBA B. ovis* was not able to express VirB8, whereas WT *B. ovis* maintained the expression of T4SS proteins. For consistent Western blot analyses, protein concentrations were confirmed by staining SDS-PAGE gel of the sample lysate with Comassie brilliant blue (Figure 4A-C).

These findings showed that the expression of T4SS-encoded proteins is independent of low pH in *B. ovis*, but is dependent on the putative ABC transporter. In close agreement with the bacteriology and confocal microscopy results, these data suggested that Δ*abcBA B. ovis* did not survive intracellularly due to a lack of T4SS expression.

### The AbcEDCBA transporter regulates the Brucella ovis T4SS at a post transcriptional level

**Figure 5:**
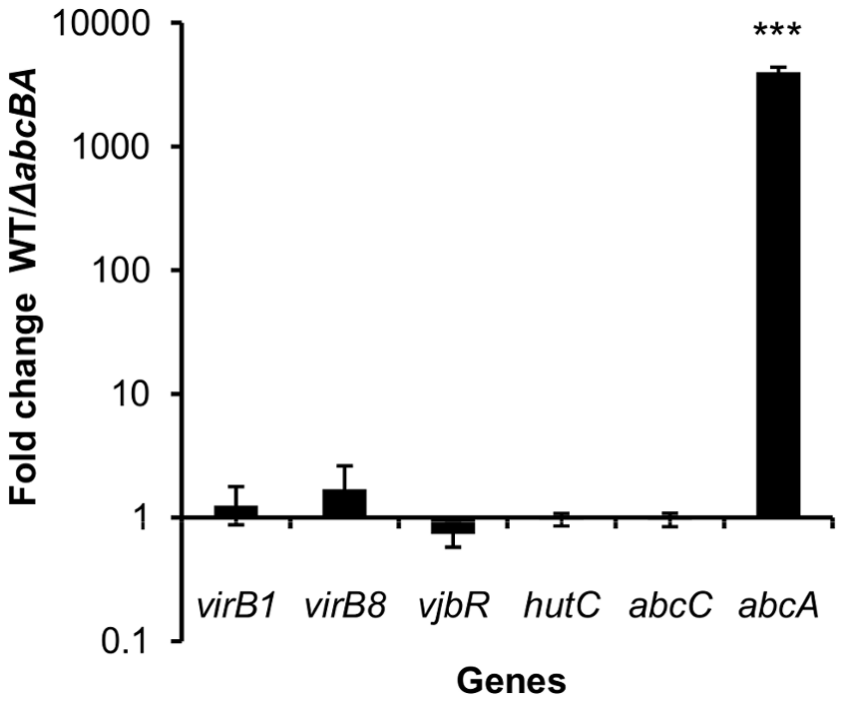
Regulation of T4SS by the ABC transporter at a post-transcriptional level in *Brucella ovis*. Real time RT-PCR of transcriptional levels of *virB1, virB8,* and regulators *vjbR and hutC* in WT *B. ovis* compared to *ΔabcBA* strain. A conserved gene (*abcC*) and a deleted gene in *ΔabcBA* (*abcA*) were used as controls. Data represent geometric mean of *B. ovis* WT fold change compared to *ΔabcBA* in three independent experiments. ***p = 3.5×10^−5^.

To determine if weak expression of T4SS-encoded proteins in *B. ovis* Δ*abcBA* was due to an effect on *virB* transcription, mRNA levels of *virB1*, *virB8* and two T4SS regulators (*hutC* and *vjbR*) were measured in *B. ovis*. Both WT and mutant strains were grown overnight in rich media (TSB with 10% FBS), under the same conditions as used for Western blot analysis, and Ct values of WT were compared to those obtained for the Δ*abcBA* mutant. Interestingly, real time RT-PCR showed that WT and Δ*abcBA B. ovis* strains had similar abundance of transcripts for *virB* genes as well as *hutC* and *vjbR* (Figure 5). This result suggests that the predicted ABC transporter does not have an impact on T4SS expression at the level of transcription or mRNA stability, but may rather act at a post-transcriptional level.

As controls, mRNA levels of a deleted gene (*abcA*) and a conserved gene upstream of the deleted region (*abcC*) were also measured. As shown in Figure 5, no significant difference on *abcC* transcription was observed between the strains, inducating that deletion of Δ*abcBA* did not affect the transcription of the *abcEDCBA* operon. As expected, WT *B. ovis* showed significantly higher Ct values of *abcA* when compared to Δ*abcBA*, which confirms the effective deletion of the transporter gene in the mutant strain.

## Discussion

Considering previous data [18], [23] and the genomic analysis, *B. ovis* ABC transporter AbcEDCBA encoded by BOPI-1 was predicted to be a peptide importer of the Opp/Tpp/Nik family. Initially, nickel uptake was evaluated during *in vitro* growth of both WT and Δ*abcBA B. ovis* strains. As shown in Figures S1, although *B. ovis* Δ*abcBA* showed limited growth in different nickel conditions, it was similar to WT *B. ovis* growth, suggesting that nickel was taken up equally by both strains. In *Brucella* spp., three main nickel importers are demonstrated: an ABC transporter *nikA-E* and two Energy Coupling Factor (ECF-type) transporters, *nikK-O* and *ure2* [45], [46]. Previous genomic study revealed that two genes from *ure2* (BOV_1316 and BOV_1319) and *nikD* (BOV_A0751) are pseudogenes in *B. ovis* [18], which may compromise the function of two nickel importers. However, ECF *nikK-O* is conserved and potentially may compensate nickel uptake in Δ*abcBA B. ovis* mutant. Therefore, we could not exclude the role of ABC transporter as nickel importer.

Additionally, ABC transporter function as peptide importer was analyzed in *Salmonella* Typhimurium by expressing *B. ovis abcA-E* locus and evaluating its sensitivity to known toxic peptides [29], [32], [47]. The complemented strain STm-comp was not able to take up the peptides tri-lysine and alafosfalin, which are expected to be imported by Tpp and Opp transporters, respectively (Figures S2). These findings imply that *B. ovis* ABC transporter probably did not work as tripeptide or as oligopeptide importer in the *Salmonella* assay, or that its specificity may be different from that of the corresponding *Salmonella* transporters. Moreover, WT and Δ*abcBA B. ovis* strains were not susceptible to the highest dose of toxic peptides in nutrient-limited media. *B. ovis* resistance against tri-lysine might result from a protective effect of outer membrane proteins, as rough strains are highly resistant to antimicrobial cationic peptides [48]. With these findings, it was not possible to conclude which specific substrate AbcEDCBA transports, so additional metabolic and proteomic comparative assays will be necessary to identify its function.

An additional effort in this study to understand the role of the *B. ovis*-specific ABC transporter was based on the comparison of the proteomic profiles between the WT and Δ*abcBA*. Interestingly, proteomic data resulted in the identification of five ABC transporter binding proteins which were considered pseudogenes according to the currently available *B. ovis* genomic data. This finding clearly indicates that computational annotation of *B. ovis* genome needs revision, given that predicted pseudogenes are expressed and potentially functional. Another proteomic study also demonstrated annotation errors in *B. abortus* database, after identifying four proteins encoded by pseudogenes [49].

Surprisingly, the Δ*abcBA B. ovis* strain had lower expression of 10 distinct ABC system binding proteins, including glycine betaine/L-proline (ProX), oligopeptides, D-ribose (RbsB), D-xylose (XylF), and glicerol (UgpB) importers (Table 4). This suggests that the ATPase deletion of a predicted peptide importer compromises other ABC systems and, consequently, restricts nutritional uptake in the Δ*abcBA* strain. Additionally, the Δ*abcBA B. ovis* strain had decreased expression of membrane protein Omp31 (BOV_1156), and Cu-Zn Sod (SodC, BOV_A0659) and Fe-Mn Sod (SodB, BOV_0567) antioxidants, which are well characterized virulence factors in *Brucella* spp. [50], [51], [52], [53]. Previous studies have shown that Omp31 is not required for *B. ovis* intracellular survival [54], [55], but it is immunogenic in rough *Brucella* spp. species [50]. Other proteomic studies demonstrated reduced Omp31 expression in a *B. melitensis virB* mutant strain [56], [57].

Both SodC and SodB are conserved in the *Brucella* genus and essential for intracellular survival by evading the respiratory burst within phagocytes [51]. In *B. abortus,* a mutant lacking SodC is attenuated in macrophages and in mouse model [52]. Therefore, decreased Sod expression in the Δ*abcBA B. ovis* strain may be, at least partially, responsible for the attenuation of this strain [19], [58].

*B. ovis* AbcEDCBA was previously shown to play a role in pathogenesis of *B. ovis*, as Δ*abcBA B. ovis* strain was attenuated in a mouse model as early as one day post infection and in mouse peritoneal macrophages at 12 µhpi [19]. A recent study also characterized the kinetics of Δ*abcBA* infection in sexually mature rams. The mutant lacking ABC transporter was not excreted in semen and urine of infected rams, although it induced a similar lymphocytic proliferative response when compared to WT *B. ovis* [58]. Considering that Δ*abcBA* was attenuated in both murine model and in the natural host, bacterial infection and trafficking were characterized in HeLa cells, to understand how ABC transporter contributes to *B. ovis* intracellular survival and replication. Epithelial cell lines differ from phagocytic cells due to lack of bactericidal activity against *Brucella* sp. [36], [41], which allows for the study of infection and trafficking of severely attenuated mutants, including Δ*abcBA B. ovis*.

Even though *B. ovis* is a naturally rough and non zoonotic species [2], it was able to establish infection and successfully replicate in HeLa cells. Interestingly, the Δ*abcBA* mutant strain showed lower colonization in HeLa cells at 24 µhpi and the infection was controlled until 48 hours, although both strains demonstrated similar internalization (Figure 2). The kinetics of Δ*abcBA* infection was similar to that previously described in RAW macrophage cell line [19], which supports the notion that predicted ABC importer is crucial for *B. ovis* intracellular survival, even in the absence of macrophage bactericidal mechanisms.

Trafficking of classical pathogenic species of *Brucella* spp. is well described in both phagocytic and nonphagocytic cells [17], [36], [38], [41], [59]–[61]. However, few studies have characterized the replication and intracellular trafficking of naturally rough *Brucella* spp. [62], [63]. This is the first work illustrating *B. ovis* trafficking in HeLa cells by confocal microscopy, which demonstrate bacterial escape from LAMP-1^+^ compartment and intracellular replication at later time points. As shown in Figure 3, *B. ovis* intracellular trafficking was very similar to classical smooth *Brucella* spp. [17], [41], with early interaction of BCV with lysosome (LAMP1^+^), followed by exclusion of LAMP1 and bacterial replication. Notably, *B. ovis* showed a later evasion of lysosome fusion only seen at 48 µhpi (Figure 4B), and not as early as 24 hours, as described for *B. abortus* [41].

Compared to WT *B. ovis*, the Δ*abcBA* mutant strain remained within a LAMP-1^+^ compartment at all time points and, consequently, was not able to survive in HeLa cells (Figure 4). The trafficking defect observed for Δ*abcBA B. ovis* was identical to that previously described for *Brucella* spp. mutants with a non-functional T4SS, as they lose the capacity of excluding LAMP-1 after the initial fusion with the phagolysosomal compartment [7], [17], [41]. Exclusively in *B. ovis*, T4SS seems to have a critical role not only for persistent infection, but also for establishing early infection in mice and in peritoneal macrophages [16]. In smooth *B. abortus,* however, lack of T4SS does not interfere with early infection in the mouse, showing phenotype similar as the WT strain until five days post infection [9], [10], [13], [16]. In agreement with our findings, previous studies demonstrated that *B. ovis* Δ*abcBA and* Δ*virB2* mutants have identical phenotypes in both mouse model and macrophages [16], [19]. Interestingly, this study shows that the *B. ovis* Δ*abcBA* mutant was unable to express VirB proteins in either rich media or acid minimal media that mimics the early BCV environment (Figure 4). Therefore, it is likely that inactivation of AbcEDCBA results in decreased expression of T4SS and, consequently, impairment of *B. ovis* trafficking and replication within cells.

Early lysosome interaction during *Brucell*a sp. intracellular trafficking is necessary for acidification and maturation of BCV, which is essential for bacterial survival, by inducing T4SS expression [17], [38], [64]. The expression of *virB*-encoded proteins in classical *Brucella* sp. is induced by low pH and poor nutritional conditions, which are observed during the initial phase of intracellular trafficking (approximately 5 hours) [38], [42], [44], [65]. Conversely, WT *B. ovis* differed from all other *Brucella* species, due to *in vitro* expression of VirB proteins in both acid and rich neutral media (Figure 4). This showed an exclusive mechanism for T4SS regulation in *B. ovis*, which was independent of an acidic environment.

Previous studies identified protein regulators of T4SS in *Brucella* spp., including histidine pathway HutC and a quorum sensing VjbR. Both regulators have an important role by directly binding the *virB* promoter and actively inducing *virB* transcription in a nutrient-poor environment, with low pH, and in presence of urocanic acid [38], [39], [43], [44]. Considering that *B. ovis* also expressed T4SS in rich neutral media and that lack of VirB expression in Δ*abcBA B. ovis* was observed at any *in vitro* condition (Figure 4), we analyzed whether the transcription of the these two regulators or of the *virB* genes was affected in the Δ*abcBA* mutant. However, the reduced abundance of VirB proteins was not explained by defects in transcription or mRNA stability, since Δ*abcBA and* WT *B. ovis* showed similar mRNA levels of *hutC*, *vjbR, virB1,* and *virB8* genes (Figure 5). Post transcriptional regulation of T4SS proteins have been previously described in *B*. *abortus* [36]. In this species, the expression of VjbR and VirB7 were induced *in vitro* by low pH and solely in the presence of urocanic acid, although similar promoter activities of *virB* and *vjbR* were noticed in different growth conditions [39]. Taken together, our data supports the notion that T4SS expression in *B. ovis* is regulated by AbcEDCBA at a post transcriptional level. One possible mechanism by which this might occur is uptake of a substrate that acts directly to affect translation of *virB* mRNA, however alternatively, uptake of the ABC transporter substrate could have an indirect effect on VirB protein levels via indirect effects on other metabolic or regulatory pathways. Further experiments will be necessary to distinguish between these possibilities and to identify the substrate of the ABC transporter.

In conclusion, the result of this work revealed that the predicted peptide ABC importer AbcEDCBA was required for *B. ovis* in vitro expression of other ABC systems and virulence proteins (including Omp31 and Sod), as well as its intracellular survival and evasion from phagosome/lysosome fusion, by interfering with the expression of T4SS-encoded proteins through a post transcriptional mechanism.

## Supporting Information

Figure S1 ***In vitro*** **growth of** ***Brucella ovis*** **wild-type and** **Δ** ***abcBA*** **strains.** (A) Trypticase soy broth (TSB) supplemented with fetal bovine serum (FBS) at 0, 2, 5, or 10% of and bacterial growth measured after 24 hours. (B) *B. ovis* WT and Δ*abcBA* growth after 48 h in standard media (TSB with 10% FBS) after adding NiSO_4_ (0.5, 1 or 2 mM) or chelating nickel and other divalent cations with EDTA (10, 25 or 50 mM). Data represent average and standard error of three independent experiments.
(TIF)

Figure S2 **In vitro growth and metabolic activity of** ***Salmonella*** **Typhimurium mutant lacking peptide transporters.** S. Typhimurium LT2 wild type (STm WT) and TT17573 mutant with non-functional peptide transporters (*oppBC tppB dpp*) growth in minimal media and tetrazolium (TTC) reduction. Data represent mean and standard deviation of triplicates in one experiment.
(TIF)

Figure S3 **Peptide uptake in** ***Salmonella*** **Typhimurium expressing** ***Brucella ovis*** **ABC transporter.** (A) Lethality assay of *S.* Typhimurium LT2 (STm WT), STm TT17573 mutant with afunctional peptide transporters (*oppBC tppB dpp*), and STm TT17573 complemented with *B. ovis* locus *abcEDCBA* (TMS14). Samples grown on minimal media plate containing 0.2 and 0.4 mg of trilysine (left column) or 0.5 and 1 mg of alafosfalin (right column). (B) Diameter (mm) of inhibitory growth zone of STm WT around filter disks containing trilysine or alafosfalin. (C) Expression of *B. ovis* ABC transporter in TMS14 during *in vitro* growth confirmed by anti-histidine (α-his) Western blot. Figure is representative of three independent experiments.
(TIF)

Figure S4 **Heat map and principal component analysis of differently expressed protein spots in DIGE profile.** (A) Heat map of 100 spots (lines) differently expressed between Δ*abcBA* (left column) and WT (right column) *B. ovis* strains. Expression values are shown on a log scale ranging from −1.5 (down-regulated, green) to +1.5 (up-regulated, red). (B) Principal component analysis shows distribution of highly expressed (right area) and lowly expressed (left area) spots in the score plot. Spots within circle correspond to 95% of confidence interval.
(TIF)

Figure S5 **Interaction network of proteins with lower expression in** **Δ** ***abcBA Brucella ovis*** **.** The red boxes indicate lowly expressed proteins excised from gels and identified by mass spectrometry. Numbers represent the corresponding spot identification. Asterisk indicates identified protein annotated as pseudogene in the *B. ovis* genome.
(TIF)

Figure S6 **Interaction network of proteins with higher expression in** **Δ** ***abcBA Brucella ovis*** **.** The red boxes indicate highly expressed proteins excised from gels and identified by mass spectrometry. Numbers represent the corresponding spot identification.
(TIF)

Figure S7 **Expression of VirB8 and VirB11 by** ***Brucella ovis*** **and** ***Brucella abortus*** **grown under rich and neutral media.** *B. ovis* and *B. abortus* were cultured in blood agar plates (B) or TSA with 10% hemoglobin (H), and expression of VirB8 and VirB11 was evaluated by Western blot.
(TIF)

Table S1 **Proteins with lower expression in** ***Brucella ovis*** **Δ** ***abcBA*** **identified by mass spectrometry.**
(DOC)

Table S2 **Proteins with greater expression in** ***Brucella ovis*** **Δ** ***abcBA*** **identified by mass spectrometry.**
(DOC)
