# Supplementary figures and images for: The Predicted ABC Transporter AbcEDCBA Is Required for Type IV Secretion System Expression and Lysosomal Evasion by Brucella ovis
Source: PLoS One. 2014 Dec 4;9(12):e114532. doi: 10.1371/journal.pone.0114532 (PMC4256435; doi:10.1371/journal.pone.0114532)

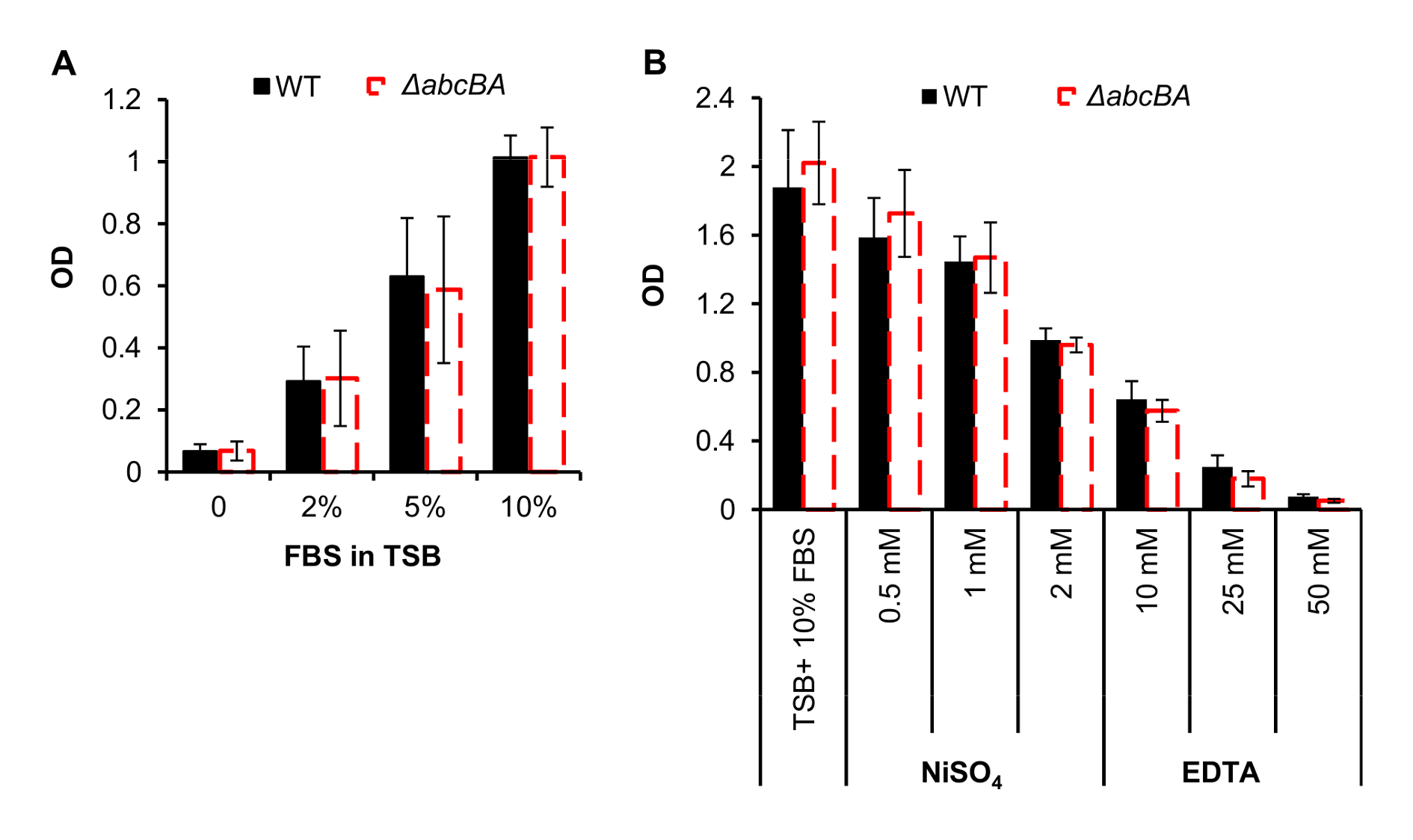

Supplement: Figure S1 — In vitro growth of Brucella ovis wild-type and Δ abcBA strains. (A) Trypticase soy broth (TSB) supplemented with fetal bovine serum (FBS) at 0, 2, 5, or 10% of and bacterial growth measured after 24 hours. (B) B. ovis WT and ΔabcBA growth after 48 h in standard media (TSB with 10% FBS) after adding NiSO4 (0.5, 1 or 2 mM) or chelating nickel and other divalent cations with EDTA (10, 25 or 50 mM). Data represent average and standard error of three independent experiments. (TIF) [file pone.0114532.s001.tif]

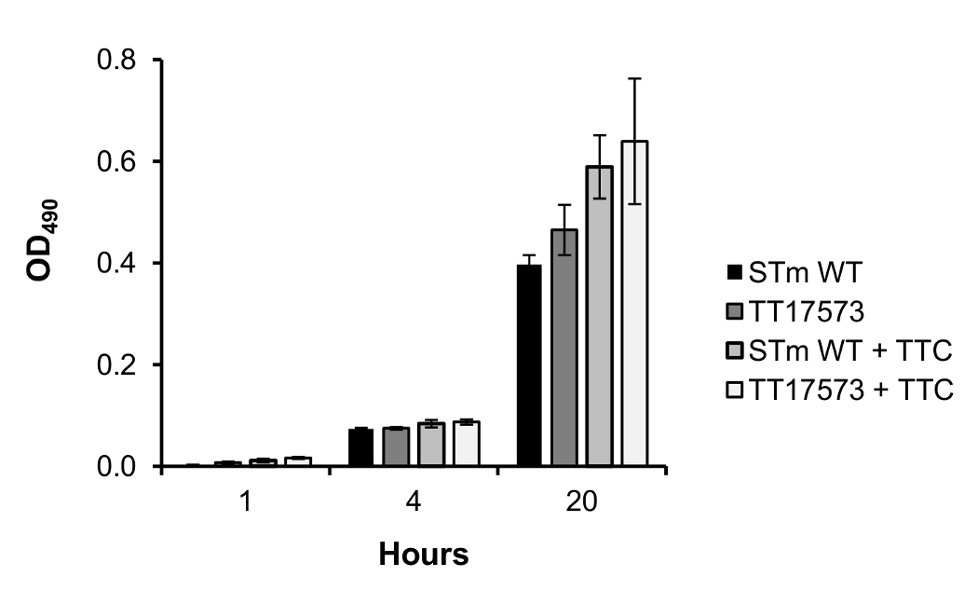

Supplement: Figure S2 — In vitro growth and metabolic activity of Salmonella Typhimurium mutant lacking peptide transporters. S. Typhimurium LT2 wild type (STm WT) and TT17573 mutant with non-functional peptide transporters (oppBC tppB dpp) growth in minimal media and tetrazolium (TTC) reduction. Data represent mean and standard deviation of triplicates in one experiment. (TIF) [file pone.0114532.s002.tif]

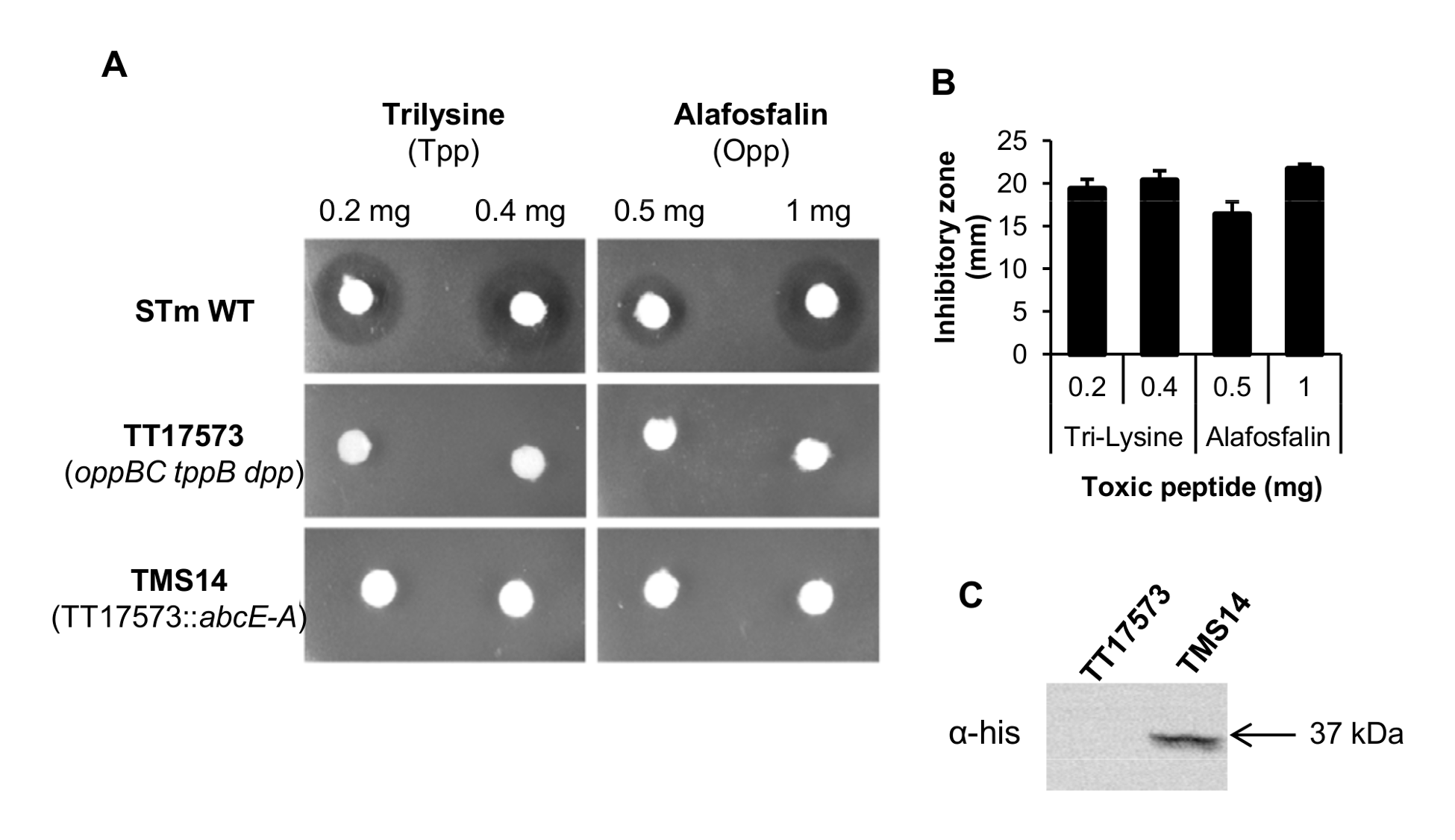

Supplement: Figure S3 — Peptide uptake in Salmonella Typhimurium expressing Brucella ovis ABC transporter. (A) Lethality assay of S. Typhimurium LT2 (STm WT), STm TT17573 mutant with afunctional peptide transporters (oppBC tppB dpp), and STm TT17573 complemented with B. ovis locus abcEDCBA (TMS14). Samples grown on minimal media plate containing 0.2 and 0.4 mg of trilysine (left column) or 0.5 and 1 mg of alafosfalin (right column). (B) Diameter (mm) of inhibitory growth zone of STm WT around filter disks containing trilysine or alafosfalin. (C) Expression of B. ovis ABC transporter in TMS14 during in vitro growth confirmed by anti-histidine (α-his) Western blot. Figure is representative of three independent experiments. (TIF) [file pone.0114532.s003.tif]

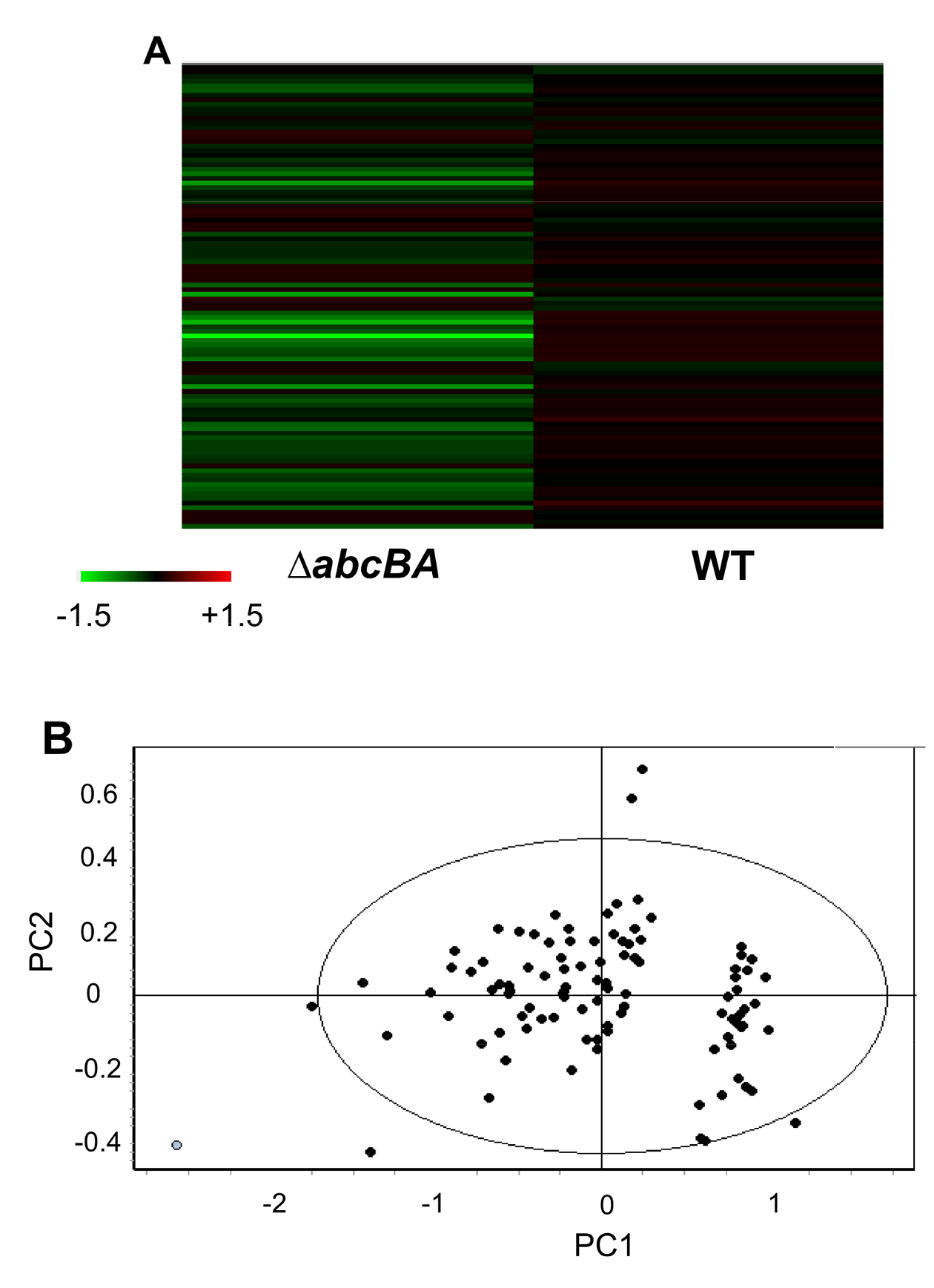

Supplement: Figure S4 — Heat map and principal component analysis of differently expressed protein spots in DIGE profile. (A) Heat map of 100 spots (lines) differently expressed between ΔabcBA (left column) and WT (right column) B. ovis strains. Expression values are shown on a log scale ranging from −1.5 (down-regulated, green) to +1.5 (up-regulated, red). (B) Principal component analysis shows distribution of highly expressed (right area) and lowly expressed (left area) spots in the score plot. Spots within circle correspond to 95% of confidence interval. (TIF) [file pone.0114532.s004.tif]

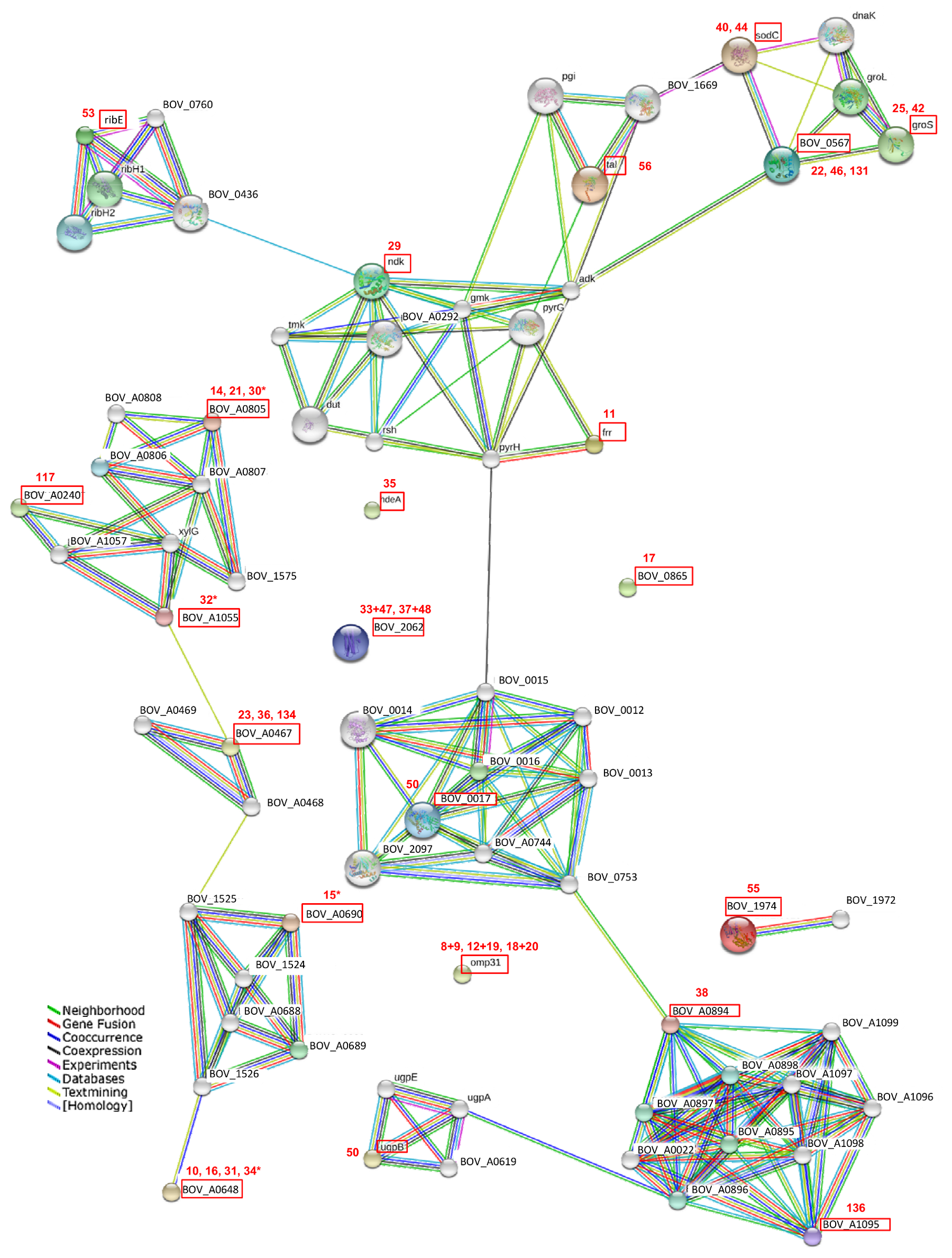

Supplement: Figure S5 — Interaction network of proteins with lower expression in Δ abcBA Brucella ovis . The red boxes indicate lowly expressed proteins excised from gels and identified by mass spectrometry. Numbers represent the corresponding spot identification. Asterisk indicates identified protein annotated as pseudogene in the B. ovis genome. (TIF) [file pone.0114532.s005.tif]

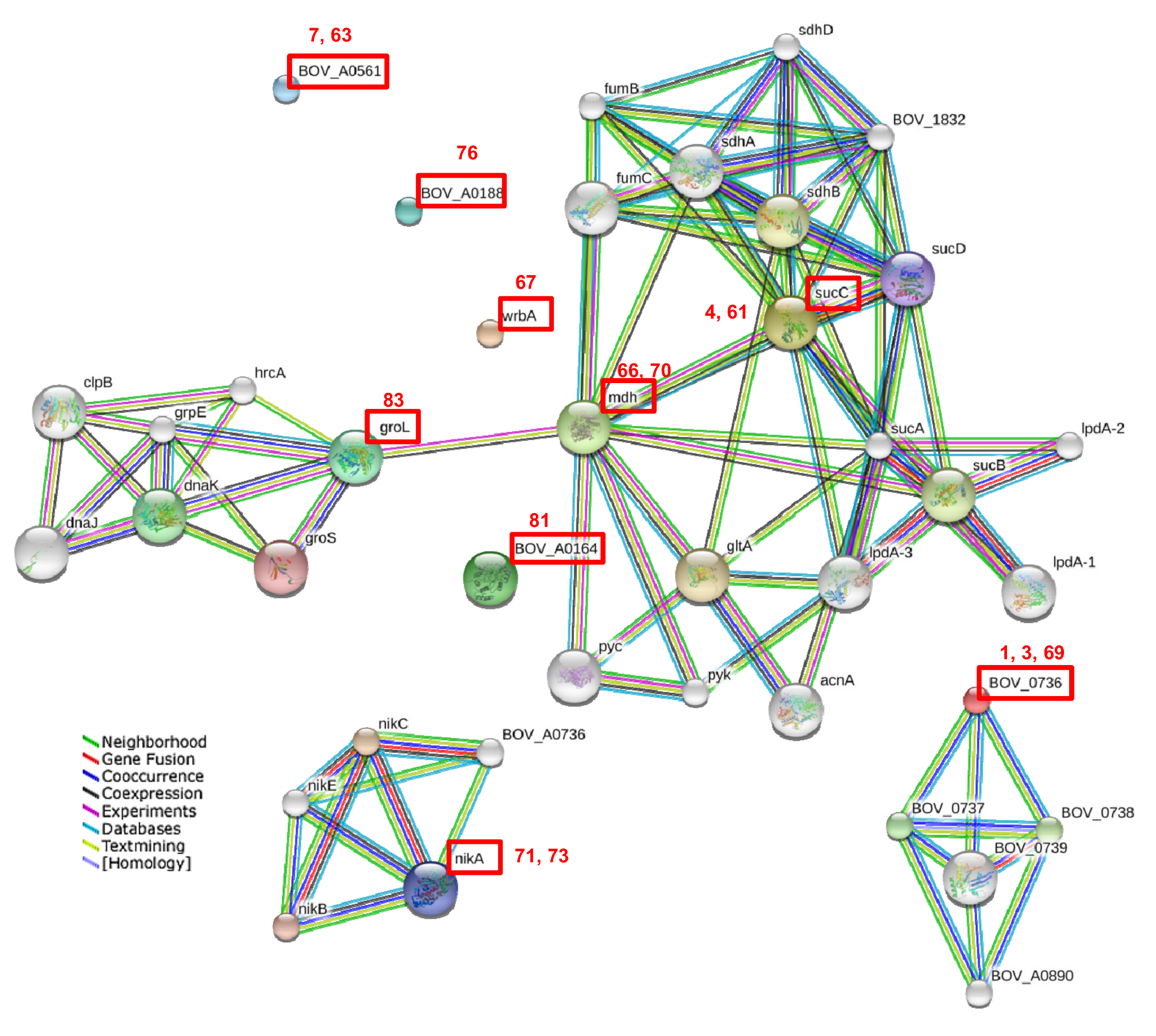

Supplement: Figure S6 — Interaction network of proteins with higher expression in Δ abcBA Brucella ovis . The red boxes indicate highly expressed proteins excised from gels and identified by mass spectrometry. Numbers represent the corresponding spot identification. (TIF) [file pone.0114532.s006.tif]

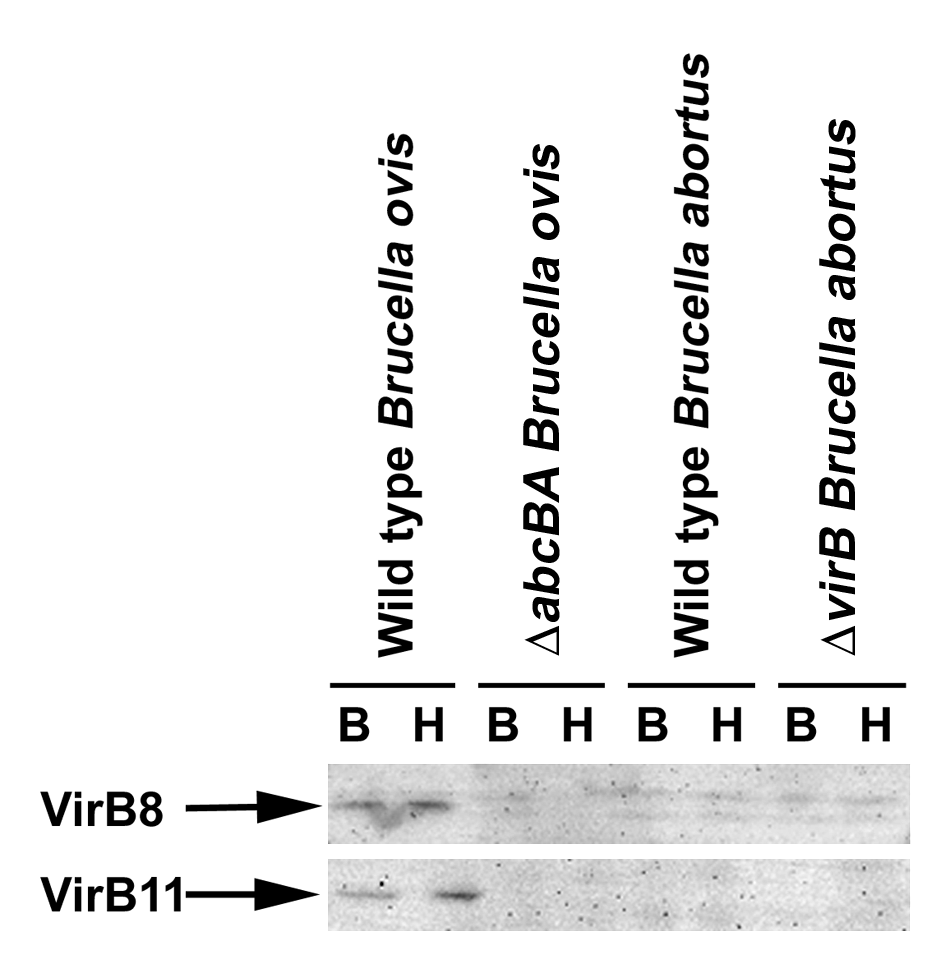

Supplement: Figure S7 — Expression of VirB8 and VirB11 by Brucella ovis and Brucella abortus grown under rich and neutral media. B. ovis and B. abortus were cultured in blood agar plates (B) or TSA with 10% hemoglobin (H), and expression of VirB8 and VirB11 was evaluated by Western blot. (TIF) [file pone.0114532.s007.tif]
