# Supplementary material for: The Predicted ABC Transporter AbcEDCBA Is Required for Type IV Secretion System Expression and Lysosomal Evasion by Brucella ovis
Source: PLoS One. 2014 Dec 4;9(12):e114532. doi: 10.1371/journal.pone.0114532 (PMC4256435; doi:10.1371/journal.pone.0114532)
Supplement: Table S1 — Proteins with lower expression in Brucella ovis Δ abcBA identified by mass spectrometry. (DOC) [file pone.0114532.s008.doc]

Table S1. Proteins with lower expression in *Brucella ovis ∆abcBA* identified by mass spectrometry.

| **Spot^a^** | **Gene ID^b^** | **Function^c^** | **Score** | **%^d^** | **pI^e^ (pred/exp)** | **MW^f^ (pred/exp)** | **Peptide sequence** |
| --- | --- | --- | --- | --- | --- | --- | --- |
| 8+9 | BOV_1156 | 31 kDa immunogenic protein | 280 | 17 | 5.49/5.78 | 33/30.5 | 100-114 R.LLATLYPETIHIVAR.K |
|  |  |  |  |  |  |  | 130-144 R.VSLDEPGSGTIVDAR.I |
|  |  |  |  |  |  |  | 145-158 R.IVLEAYGLTEDDIK.A |
|  |  |  |  |  |  |  | 159-170 K.AEHLKPGPAGER.L |
| 10 | BMEII0590* | ABC transporter sugar binding protein | 171 | 14 | 4.97/4.90 | 43.3/39.3 | 11-32 K.QNVEVLHWWTSGGEASALEVLK.K |
|  |  |  |  |  |  |  | 63-80 R.VTAGNAPTAVQMLGFDIR.D |
|  |  |  |  |  |  |  | 221-230 R.TYVDDNFSGR.D |
|  |  |  |  |  |  |  | 263-272 K.KPGEDFVCMR.Y |
| 11 | BOV_1116 | ribosome recycling factor | 164 | 19 | 6.3/6.67 | 20.7/23.8 | 2-12 M.SDAFDINDLKR.R |
|  |  |  |  |  |  |  | 85-100 R.DSGLGLNPITDGMTLR.I + Oxidação (M) |
|  |  |  |  |  |  |  | 117-126 K.IAHQYAEQGR.I |
| 12+19 | BOV_1156 | 31 kDa immunogenic protein | 123 | 9 | 5.49/5.54 | 33/30.5 | 100-114 R.LLATLYPETIHIVAR.K |
|  |  |  |  |  |  |  | 145-158 R.IVLEAYGLTEDDIK.A |
| 14 | BMEII0435* | D-ribose-binding periplasmic protein precursor | 155 | 14 | 5.6/4.82 | 31/28.2 | 129-144 K.YVELFGAPSDNNAATR.S |
|  |  |  |  |  |  |  | 216-230 K.VGGFDGSPDAIAAIK.A |
|  |  |  |  |  |  |  | 269-278 K.QLFDCILITK.D |
| 15 | BMEII0550* | glycine betaine/L-proline ABC transporter substrate-binding protein | 168 | 16 | 5.57/5.27 | 31.8/29.5 | 59-75 K.VELVQTDVAPLYQGVSR.G |
|  |  |  |  |  |  |  | 141-156 K.LKGEIQGIDPGAGLTR.L |
|  |  |  |  |  |  |  | 217-230 K.GALGGAEHIDAVAR.K |
| 16 | BMEII0590* | sugar binding protein | 101 | 5 | 4.97/4.84 | 43.3/39.9 | 103-113 K.VIPAPLQEFAK.Y |
|  |  |  |  |  |  |  | 221-230 R.TYVDDNFSGR.D |
| 17 | BOV_0865 | hypothetical protein | 80 | 5 | 5.07/5.31 | 24.8/29 | 153-166 K.ISGLDGEAEAFLAR.E |
| 18+20 | BOV_1156 | 31 kDa immunogenic protein | 89 | 9 | 5.49/6.05 | 33/30.9 | 100-114 R.LLATLYPETIHIVAR.K |
|  |  |  |  |  |  |  | 145-158 R.IVLEAYGLTEDDIK.A |
| 21 | BMEII0435* | D-ribose-binding periplasmic protein precursor | 99 | 10 | 5.6/4.75 | 31/28.4 | 129-144 K.YVELFGAPSDNNAATR.S |
|  |  |  |  |  |  |  | 216-230 K.VGGFDGSPDAIAAIK.A |
| 22 | BOV_0567 | superoxide dismutase, Fe-Mn family | 78 | 15 | 5.83/6.10 | 22.5/23 | 2-21 M.AFELPALPYDYDALAPFMSR.E + Oxidação (M) |
|  |  |  |  |  |  |  | 100-110 K.AFDSDLGGYDK.F |
| 23 | BOV_A0467 | oligopeptide ABC transporter substrate-binding protein | 162 | 8 | 5.09/5.02 | 59/52 | 227-238 K.IDTVNWMPFEDR.S |
|  |  |  |  |  |  |  | 309-317 R.EFMADEVWR.G + Oxidação (M) |
|  |  |  |  |  |  |  | 341-351 K.LDFADEDILDR.E |
|  |  |  |  |  |  |  | 361-374 K.EAGVEPNTLSVTLR.Y |
| 25 | BOV_A0178 | co-chaperonin GroES | 122 | 16 | 5.83/5.56 | 10.3/12.5 | 36-51 K.EKPQEGEVVAAGAGAR.D + Glu->pyro-Glu (N-term E) |
| 29 | BOV_0685 | nucleoside diphosphate kinase | 91 | 15 | 5.27/5.52 | 15.2/17.1 | 6-16 R.TFSMIKPDATR.R + Oxidação (M) |
|  |  |  |  |  |  |  | 45-55 R.REAEGFYAVHK.D |
| 30 | BMEII0435* | D-ribose-binding periplasmic protein precursor | 75 | 10 | 5.6/4.67 | 31/28.2 | 129-144 K.YVELFGAPSDNNAATR.S |
|  |  |  |  |  |  |  | 216-230 K.VGGFDGSPDAIAAIK.A |
| 31 | BMEII0590* | sugar binding protein | 85 | 5 | 4.97/4.79 | 43.3/40.3 | 103-113 K.VIPAPLQEFAK.Y |
|  |  |  |  |  |  |  | 221-230 R.TYVDDNFSGR.D |
| 32 | BMEII0146* | D-xylose-binding periplasmic protein precursor | 82 | 8 | 5.05/5.03 | 34.2/34.1 | 30-40 R.DRDYFIAAAEK.L |
|  |  |  |  |  |  |  | 102-118 R.LILNADIDAYISFDNER.V |
| 33+47 | BOV_2062 | DNA starvation/stationary phase protection protein Dps | 160 | 28 | 5.25/5.34 | 18.3/18.5 | 48-62 K.GPQFIAVHEMLDGFR.A + Oxidação (M) |
|  |  |  |  |  |  |  | 63-75 R.AELDDHVDTIAER.A |
|  |  |  |  |  |  |  | 130-148 K.DADDAGDDDTADIFTAASR.S |
| 34 | BMEII0590* | sugar binding protein | 170 | 7 | 4.97/5.02 | 43.3/39.7 | 103-113 K.VIPAPLQEFAK.Y |
|  |  |  |  |  |  |  | 221-230 R.TYVDDNFSGR.D |
|  |  |  |  |  |  |  | 263-272 K.KPGEDFVCMR.Y |
| 35 | BOV_A0312 | Acid stress chaperone HdeA | 121 | 21 | 6.59/5.82 | 12.5/12.5 | 71-95 K.GKEEDAVIDVDGIETVTPAIIEACK.Q |
| 36 | BOV_A0467 | oligopeptide ABC transporter substrate-binding protein | 133 | 6 | 5.09/4.98 | 59/52 | 227-238 K.IDTVNWMPFEDR.S |
|  |  |  |  |  |  |  | 309-317 R.EFMADEVWR.G |
|  |  |  |  |  |  |  | 361-374 K.EAGVEPNTLSVTLR.Y |
| 37+48 | BOV_2062 | DNA starvation/stationary phase protection protein Dps | 266 | 36 | 5.25/5.58 | 18.3/18 | 48-62 K.GPQFIAVHEMLDGFR.A + Oxidação (M) |
|  |  |  |  |  |  |  | 63-75 R.AELDDHVDTIAER.A |
|  |  |  |  |  |  |  | 130-148 K.DADDAGDDDTADIFTAASR.S |
|  |  |  |  |  |  |  | 153-165 K.ALWFLEAHVQESN.- |
| 38 | BOV_A0894 | ABC transporter periplasmic amino acid-binding protein | 120 | 6 | 5.31/5.35 | 43.3/39.7 | 237-249 K.QAAEFGIVAGGQR.L + Gln->pyro-Glu (N-term Q) |
|  |  |  |  |  |  |  | 332-344 K.KLHEMPVEDVFAR.H + Oxidação (M) |
| 40 | BOV_A0659 | superoxide dismutase, Cu-Zn | 76 | 23 | 6.24/6.70 | 18.2/16.1 | 38-54 K.EVGTVVISEAPGGLHFK.V |
|  |  |  |  |  |  |  | 144-167 R.SLMIHVGGDNYSDKPEPLGGGGAR.F + Oxidação (M) |
| 42 | BOV_A0178 | co-chaperonin GroES | 92 | 16 | 5.83/5.43 | 10.3/12.6 | 36-51 K.EKPQEGEVVAAGAGAR.D |
| 44 | BOV_A0659 | superoxide dismutase, Cu-Zn | 79 | 23 | 6.24/6.52 | 18.2/16.3 | 38-54 K.EVGTVVISEAPGGLHFK.V |
|  |  |  |  |  |  |  | 144-167 R.SLMIHVGGDNYSDKPEPLGGGGAR.F + Oxidação (M) |
| 46 | BOV_0567 | superoxide dismutase, Fe-Mn family | 94 | 15 | 5.83/5.90 | 22.5/22.9 | 2-21 M.AFELPALPYDYDALAPFMSR.E + Oxidação (M) |
|  |  |  |  |  |  |  | 100-110 K.AFDSDLGGYDK.F |
| 50 | BOV_0017 | isovaleryl-CoA dehydrogenase | 118 | 9 | 5.28/5.47 | 41.9/41.3 | 34-43 R.NNQFPMHLWR.E + Oxidação (M) |
|  |  |  |  |  |  |  | 231-243 K.GVNVLMSGLDYER.V |
|  |  |  |  |  |  |  | 285-296 K.LADMYVTFNASR.A |
|  | BOV_A0617 | ABC transporter periplasmic glycerol-3-phosphate-binding protein | 96 | 3 |  |  | 76-90 K.GNYPETLNAGIAAFR.S |
| 53 | BOV_0761 | riboflavin synthase subunit alpha | 248 | 21 | 5.02/5.10 | 22.3/24.5 | 16-26 R.VKPLNEGVLLR.I |
|  |  |  |  |  |  |  | 63-74 R.WFEVEAWEEALR.L |
|  |  |  |  |  |  |  | 168-178 R.HSLEVTTWGER.K |
|  |  |  |  |  |  |  | 184-193 K.VNIEIDQLAR.Y |
| 55 | BOV_1974 | ABC transporter periplasmic substrate -binding protein | 102 | 13 | 5.45/4.99 | 33.2/36.2 | 64-81 K.FIYQSAQGNPATAAQIAR.Q |
|  |  |  |  |  |  |  | 239-250 K.LPLFTADTDSVK.R |
|  |  |  |  |  |  |  | 304-315 K.MGVEFPQAVIDR.A + Oxidação (M) |
| 56 | BOV_1738 | putative translaldolase | 72 | 4 | 5.69/6.0 | 23.6/24.4 | 149-157 U R.TIYDNYDFR.T |
| 117 | BOV_A0240 | putative sugar ABC transporter periplasmic sugar-binding protein | 145 | 10 | 5.37/5.27 | 36.3/36.4 | 109-120 K.VIAYDRPIPDAK.S |
|  |  |  |  |  |  |  | 121-134 K.SDFYVSFDNEAIGK.S |
|  |  |  |  |  |  |  | 200-210 K.AQQWASGQITR.F |
| 131 | BOV_0567 | superoxide dismutase, Fe-Mn family | 108 | 15 | 5.83/5.12 | 22.5/39.6 | 2-21 M.AFELPALPYDYDALAPFMSR.E |
|  |  |  |  |  |  |  | 100-110 K.AFDSDLGGYDK.F |
| 134 | BOV_A0467 | oligopeptide ABC transporter substrate-binding protein | 100 | 7 | 5.09/5.08 | 59/51.4 | 274-286 R.LAPYLGIYYVDIK.G |
|  |  |  |  |  |  |  | 341-351 K.LDFADEDILDR.E |
|  |  |  |  |  |  |  | 361-374 K.EAGVEPNTLSVTLR.Y |
| 136 | BOV_A1095 | ABC transporter periplasmic amino acid binding protein | 228 | 15 | 5.46/5.29 | 43.7/40.9 | 91-103 K.IYDNQSNVNLSVR.Q |
|  |  |  |  |  |  |  | 138-153 R.IPMVQGGGASDEIYSR.N + Oxidação (M) |
|  |  |  |  |  |  |  | 183-204 K.VTNVALVYADDSFDVSVADGTR.K |
|  |  |  |  |  |  |  | 267-280 K.LYSFTVGVPTEDFR.K |

^a^ The numbers correspond to the specific spots as indicated in Figure 5.

^b^ Genes correspond to *Brucella ovis* genome database according to NCBI.

^c^ Predicted protein function according to UniProt and NCBI.

^d^ Percentage of coverage

^e^ Isoelectric point

^f^ Molecular weight (kDa)

* Genes correspond to *Brucella melitensis* 16M database and were annotated as pseudogenes in *Brucella ovis*.
