# Supplementary material for: The Predicted ABC Transporter AbcEDCBA Is Required for Type IV Secretion System Expression and Lysosomal Evasion by Brucella ovis
Source: PLoS One. 2014 Dec 4;9(12):e114532. doi: 10.1371/journal.pone.0114532 (PMC4256435; doi:10.1371/journal.pone.0114532)
Supplement: Table S2 — Proteins with greater expression in Brucella ovis Δ abcBA identified by mass spectrometry. (DOC) [file pone.0114532.s009.doc]

Table S2. Proteins with greater expression in *Brucella ovis ∆abcBA* identified by mass spectrometry.

| **Spot^a^** | **Gene ID^b^** | **Function^c^** | **Score** | **%^d^** | **pI^e^ (pre/exp)** | **MW^f^ (pre/exp)** | **Peptide sequence** |
| --- | --- | --- | --- | --- | --- | --- | --- |
| 1 | BOV_0736 | ABC transporter periplasmic amino acid-binding protein | 318 | 18 | 5.3/5.18 | 37.3/32 | 54–65 K.GEWSGFDVDYCR.A |
|  |  |  |  |  |  |  | 89–102 R.FTALQSGEVDVLIR.N |
|  |  |  |  |  |  |  | 181-192 K.LEEANAAYDSGR.C |
|  |  |  |  |  |  |  | 193-207 R.CDAYTTDQSSLYGVR.L |
|  |  |  |  |  |  |  | 303-313 K.GVGNYGELFER.N |
| 3 | BOV_0736 | ABC transporter periplasmic amino acid-binding protein | 158 | 10 | 5.3/5.06 | 37.3/32 | 54–65 K.GEWSGFDVDYCR.A |
|  |  |  |  |  |  |  | 89–102 R.FTALQSGEVDVLIR.N |
|  |  |  |  |  |  |  | 303–313 K.GVGNYGEIFER.N |
| 4 | BOV_1855 | succinyl-CoA synthetase beta chain | 83 | 5 | 4.9/5.08 | 42.6/44.3 | 99-108 R.ELYLSLLVNR.E + Glu->pyro-Glu (N-term E) |
|  |  |  |  |  |  |  | 208-217 K.VSFDNNALFR.H |
| 7 | BOV_A0561 | metal-dependent hydrolase | 186 | 33 | 5.58/6.00 | 25.3/26.6 | 43-65 R.GVTHIALTHGHGDHVGDTVAIAR.E |
|  |  |  |  |  |  |  | 66-88 R.EHGATVIANADLASWLGSQGVEK.L |
|  |  |  |  |  |  |  | 174-187 R.FTMGGAVAALACQR.Y |
|  |  |  |  |  |  |  | 188-207 R.YFNFNSVLPCHYASFPIIDR.T |
| 61 | BOV_1855 | succinyl-CoA synthetase subunit beta | 162 | 8 | 4.9/5.01 | 42.6/44 | 84-94 R.LYIEDGADIER.E |
|  |  |  |  |  |  |  | 95-104 R.ELYLSILIDR.S |
|  |  |  |  |  |  |  | 204-213 K.VSFDNNALFR.H |
| 63 | BOV_A0561 | metal-dependent hydrolase | 146 | 16 | 5.58/5.83 | 25.3/26.6 | 25-34 K.ITWLGHAAFR.V |
|  |  |  |  |  |  |  | 196-209 R.FTMGGAVAGLACQR.Y + Oxidation (M) |
|  |  |  |  |  |  |  | 210-229 R.YFNFNSVLPCHYASFPIIDR.T |
| 66 | BOV_1856 | malate dehydrogenase | 95 | 12 | 5.24/5.55 | 33.8/38 | 60-83 K.FTGANDYAAIEGADVVIVTAGVPR.K |
|  |  |  |  |  |  |  | 253-267 R.VLPVAAQLSGQYGVK.D |
| 67 | BOV_1014 | NAD(P)H dehydrogenase (quinone) | 320 | 30 | 5.85/6.32 | 21.4/25.5 | 37-48 K.RVPELVPEEVAK.A |
|  |  |  |  |  |  |  | 54-79 K.IDQEAPIATPGELADYDAIIIGTATR.Y |
|  |  |  |  |  |  |  | 89-100 K.NFLDQTGGLWAK.G |
|  |  |  |  |  |  |  | 174-184 R.QPSAQELDDAR.F + Gln->pyro-Glu (N-term Q) |
| 69 | BOV_0736 | amino acid ABC transporter substrate-binding protein | 201 | 18 | 5.3/5.31 | 37.3/32.2 | 54–65 K.GEWSGFDVDYCR.A |
|  |  |  |  |  |  |  | 89–102 R.FTALQSGEVDVLIR.N |
|  |  |  |  |  |  |  | 181-192 K.LEEANAAYDSGR.C |
|  |  |  |  |  |  |  | 193-207 R.CDAYTTDQSSLYGVR.L |
|  |  |  |  |  |  |  | 303-313 K.GVGNYGELFER.N |
| 70 | BOV_1856 | malate dehydrogenase | 209 | 16 | 5.24/ 5.29 | 33.8/38.4 | 60-83 K.FTGANDYAAIEGADVVIVTAGVPR.K |
|  |  |  |  |  |  |  | 186-199 R.YSTVAGIPLPDLVK.M |
|  |  |  |  |  |  |  | 253-267 R.VLPVAAQLSGQYGVK.D |
| 71 | BOV_A0754 | nickel ABC transporter substrate binding protein | 133 | 7 | 58.3/53.1 | 5.52/5.82 | 97-109 K.FSNGEVFDAAAVK.A |
|  |  |  |  |  |  |  | 110-121 K.ANIDTVLQNRPR.H |
|  |  |  |  |  |  |  | 455-471 K.IDAEIGQVLVSTDETAR.Q |
| 73 | BOV_A0754 | nickel ABC transporter substrate binding protein | 114 | 7 | 58.3/52.2 | 5.52/5.70 | 110-121 K.ANIDTVLQNRPR.H |
|  |  |  |  |  |  |  | 397-408 K.LTGEEESSIYAR.Q |
|  |  |  |  |  |  |  | 455-471 K.IDAEIGQVLVSTDETAR.Q |
| 76 | BOV_A0188 | zinc protease | 87 | 5 | 49.1/44.6 | 5.07/4.88 | 166-177 R.QQIVAGIEAAQR.N + Gln->pyro-Glu (N-term Q) |
|  |  |  |  |  |  |  | 187-199 K.FAEVLYGNHPYAR.D |
| 81 | BOV_A0164 | aldo/keto reductase family oxidoreductase | 304 | 11 | 39.9/29.7 | 8.76/6.51 | 158-170 K.LWNSDQGYESTLK.A |
|  |  |  |  |  |  |  | 262-276 K.HDIATEAWSPLGQGK.L |
|  |  |  |  |  |  |  | 301-312 R.WHIETGNIVIPK.S |
| 83 | BOV_A0177 | chaperonin GroEL | 281 | 10 | 57.5/54.4 | 5.08/5.32 | 59-75 K.EVELEDKFENMGAQMLR.E + Glu->pyro-Glu (N-term E) |
|  |  |  |  |  |  |  | 59-75 K.EVELEDKFENMGAQMLR.E + Oxidation (M) |
|  |  |  |  |  |  |  | 351-362 K.QQIEETTLDYDR.E + Gln->pyro-Glu (N-term Q) |
|  |  |  |  |  |  |  | 405-421 R.AAVEEGIVAGGGTALLR.A |
|  |  |  |  |  |  |  | 430-443 K.GVNADQEAGINIVR.R |

^a^ The numbers correspond to the specific spots as indicated in Figure 5.

^b^ Genes correspond to *Brucella ovis* genome database according to NCBI.

^c^ Predicted protein function according to UniProt and NCBI.

^d^ Percentage of coverage

^e^ Isoelectric point (predicted/ experimental)

^f^ Molecular weight in kDa (predicted/ experimental)
